# Cooperative control of IgA synthesis and secretion by MZB1 and the J chain

**DOI:** 10.3389/fimmu.2026.1744147

**Published:** 2026-04-17

**Authors:** Chaoqun Cui, Xiaoqian Feng, Qing Min, Chunhui Lu, Zichao Wen, Xin Meng, Runyun Zhang, Lulu Dong, Ji-Yang Wang

**Affiliations:** 1 Department of Immunology, School of Basic Medical Sciences, Fudan University, Shanghai, China; 2 State Key Laboratory of Genetic Engineering, School of Life Sciences, Fudan University, Shanghai, China; 3 Shanghai Sci-Tech Inno Center for Infection & Immunity, Shanghai, China; 4 Department of Infectious Diseases, Shanghai Key Laboratory of Infectious Diseases and Biosafety Emergency Response, Huashan Hospital, Fudan University, Shanghai, China

**Keywords:** IgA assembly, IgA polymerization, immunoglobulin A (IgA), joining chain (J chain), marginal zone B and B-1 cell-specific protein (MZB1), mucosal immunity

## Abstract

Immunoglobulin A (IgA) is the most abundantly produced antibody in mammals, with its secretory dimeric form playing a central role in maintaining intestinal homeostasis. Although the J chain is known to be essential for IgA transcytosis, its precise role in IgA biosynthesis and secretion is not fully understood. Here, using CRISPR/Cas9-edited J558 plasmacytoma cells, a mouse line that secretes IgA, we demonstrate that MZB1 and the J chain act sequentially to ensure proper IgA assembly. MZB1 stabilizes α-heavy chain-light chain complexes (HL complexes), thereby enabling their efficient association with the J chain, which subsequently drives rapid assembly into IgA dimers and higher-order polymers. Loss of MZB1 reduced the secretion of dimeric IgA, whereas J chain deficiency caused both excessive intracellular accumulation and secretion of HL complexes, while allowing the generation and secretion of limited amounts of monomeric IgA but completely abolishing dimer formation. Combined deficiency reproduced the additive defects of the single knockouts, confirming their cooperative function in a shared assembly pathway. *In vivo*, loss of MZB1, the J chain, or both altered IgA abundance and form, differentially affected susceptibility to DSS-induced colitis, and reshaped gut microbiota composition. These findings define a cooperative mechanism by which MZB1 and the J chain orchestrate IgA biogenesis, with MZB1 regulating quantity and the J chain determining quality, and reveal how variation in IgA form and abundance contributes to mucosal immune protection.

## Introduction

IgA is conserved across all mammalian species, including placental mammals, marsupials, and monotremes, as well as in birds (1, 2). In humans, several grams of IgA are secreted into the intestine each day, where it acts as a first-line defense at mucosal surfaces. IgA contributes to barrier protection by neutralizing toxins and viruses (3–5), limiting the access of intestinal antigens to the blood circulation (6–8) and shaping the composition of intestinal microbiota (9–14). These diverse functions are enabled by both the unique structural features of the IgA heavy chain and its capacity to form polymers. Like all antibodies, the fundamental unit of IgA is a monomer composed of two identical heavy chains (αHCs) paired with two identical light chains (LCs). These monomers can be further assembled into dimers or polymers containing the joining (J) chain, a small polypeptide that directs multimer formation (15, 16). The relative abundance of monomeric IgA (mIgA) and polymeric IgA (pIgA) varies considerably between serum and mucosal secretions and also differs across species. In both humans and mice, serum IgA is predominantly monomeric, whereas mucosal IgA largely exists as dimers (17).

J chain covalently links IgA monomers via disulfide bonds formed with the penultimate cysteine residues in their C-terminal tailpieces (tp), an 18-amino acid extension present in polymerizing isotypes such as IgA and IgM but absent from non-polymerizing Ig classes such as IgG (18–20). J chain is indispensable for the epithelial transport of dimeric IgA (dIgA), as it provides the binding site for the polymeric immunoglobulin receptor (pIgR), which mediates active transcytosis (21). Although the role of J chain in dIgA formation is well established (4, 22, 23), the precise timing and mechanism of its incorporation into IgA complexes and whether it also contributes to IgA assembly process itself remain poorly understood.

MZB1 (marginal zone B and B-1 cell-specific protein) is an endoplasmic reticulum (ER)-resident protein highly expressed in marginal zone B cells and B-1 cells, and its expression is further up-regulated during plasma cell differentiation (24). Early studies implicated MZB1 in the assembly and biosynthesis of IgM (24–27). More recently, our work identified MZB1 as a molecular chaperone of J chain that facilitates its incorporation into IgA (28). We showed that MZB1 physically interacts with the α-chain tailpiece (αtp) in a manner dependent on its penultimate cysteine residue, thereby preventing degradation of intracellular HC-LC (HL) complexes. Furthermore, MZB1 promotes J chain binding to IgA and supports the secretion of dimeric IgA. Based on these findings, we proposed a model in which MZB1 first stabilizes HL complexes, after which J chain associates to drive IgA multimerization. However, the precise molecular mechanism underlying this sequential process remains unresolved.

In the present study, we set out to dissect how MZB1 and J chain, individually and cooperatively, orchestrate IgA assembly and polymerization. Using CRISPR/Cas9-engineered J558 plasmacytoma cells, a mouse IgA-secreting cell line, we show that MZB1 promotes the generation of dIgA via stabilization of HL complexes. In contrast, J chain associates with the HL complexes and drives their assembly into dIgA for secretion. Complementary *in vivo* studies using mice deficient in MZB1, J chain, or both reveal that alterations in IgA quantity and quality critically influence susceptibility to DSS-induced colitis. While optimal protection requires sufficient high-quality IgA (namely dimeric IgA), elevated monomeric IgA also exerts protective effects, partly through reshaping the gut microbiota. Together, these findings define a sequential mechanism that ensures proper IgA assembly and highlight its importance in mucosal immune protection.

## Materials and methods

### Mice

*Mzb1^-/-^* mice on a C57BL/6 background have been described previously (28). To generate *Jchain^-/-^* mice, two guide RNAs-gRNA#1 (5’-TATAGACACACAGTTGGCCG-3’) and gRNA#2 (5’-GAACATTAGTTTTATACCAC-3’) targeting exon 1, and two guide RNAs-gRNA#3 (5’-CTACACTGGAAAGACGCACA-3’) and gRNA#4 (5’-TAGCAGTGAGTATAACTTCT-3’) targeting exon 4 of the mouse *Jchain* gene, together with Cas9 mRNA, were co-injected into fertilized mouse eggs to generate knockout offspring. Pups were genotyped and bred with wild-type mice to obtain F1 animals and subsequently age- and sex-matched littermate controls. Doubly deficient mice were obtained by crossing *Mzb1^-/-^* mice with *Jchain^-/-^* mice. All mice were maintained under specific pathogen-free conditions in the animal facility of Fudan University. Age- and sex-matched mice (9–11-week-old) were used in all experiments. Mice were euthanized by cervical dislocation following institutional and national animal welfare guidelines. All animal experiments and procedures were approved by the Animal Experiment Committee of Fudan University.

### Cell culture

Mouse plasmacytoma J558 cell line was obtained from ATCC. All the gene inactivated lines were derived from J558 cell line via CRISPR/Cas9 based gene editing. All cells were cultured in DMEM medium supplemented with 10% fetal bovine serum (FBS), 5 × 10^-5^ M 2-mercaptoethanol, 100 units/ml penicillin and 100 μg/ml streptomycin (Gibco).

### Establishment of MZB1 or J chain deficient J558 cells

In order to establish MZB1 or J chain deficient cell line, the gRNA (5′-GAACCAGATGAAGCGTCTCA-3′) targeting DNA within the third exon of the *Mzb1* gene and gRNA (5′-GGTGGAAGGGATGATCCTAG-3′) targeting the second exon of *Jchain* gene were designed by online software (http://www.crispr-cas.org/), which predicted high-specificity and protospacer adjacent motif target sites in the mouse exome. Construction of lentiCRISPR vector, generation of lentivirus, virus transduction into J558 cells, and selection of correctly targeted clones were performed essentially as described (29). WT-#3 (positive control) and #23 (negative control) clones were derived from J558 cells transfected with empty or *Jchain*-targeting lentiCRISPR vectors, respectively, and were used across all knockout experiments.

### Construction of expression vectors for MZB1 and J chain

The expression vector for MZB1 (pMX-MZB1-IRES-GFP) has been described previously (28). For the amplification of mouse *Jchain* cDNA, the high-fidelity KOD-plus polymerase (TOYOBO) was used to minimize PCR errors. The amplification was performed with primers 5’-CCGGAATTCGCCACCATGAAGACCCACCTGCTTCTCT-3’ and 5’- CCGCTCGAGCTAGTCAGGGTAGCAAGAATCGG-3’ and first-strand cDNA generated from total RNA of J558 cells. After verifying the sequences, *Jchain* cDNA was subcloned into pMX-IRES-hCD8α retrovirus vector. The retrovirus expressing MZB1 and/or J chain as well as their empty vector controls were produced and transfected into J558 cells.

### Cell cycle analysis

Cells were washed with cold PBS and fixed in 70% ice-cold ethanol overnight. The cells were then washed and resuspended in 0.5 ml PBS containing 100 μg/ml of RNase A (Sigma-Aldrich), and incubated for 15 min at 37 °C. Propidium iodide (Sigma-Aldrich) was added to a final concentration of 50 μg/ml, and the cell cycle was analyzed with a FACSVerse flow cytometer using the FACSuite software.

### Flow cytometry

Single-cell suspensions were prepared from freshly isolated tissues using standard procedures. Following red blood cell lysis, cells were blocked with anti-CD16/32 (Catalog no. 553142; BD Biosciences) and subsequently stained with the following fluorescent-conjugated antibodies: α-B220 (Catalog no. 552772; eBioscience), α-IgA (Catalog no. F9384; Sigma-Aldrich), α-hCD8α (Catalog no. 300912; BioLegend), α-CD45 (Catalog no. 11-0451-81; eBioscience), α-CD11b (Catalog no. 101207; Biolegend), α-F4/80 (Catalog no. 25-4801-82; eBioscience), α-Ly6G (Catalog no. 560599; BD Biosciences), α-IL-10 (Catalog no. 505026; BioLegend), α-CD79a (Catalog no. 133106; BioLegend). For live/dead cell discrimination, we used 7-AAD viability staining solution (eBioscience) or the fixable dye eFluor780 (eBioscience). For intracellular staining of IgA and CD79a, after cell surface staining, cells were fixed with IC Fixation Buffer (eBioscience) and permeabilized using Perm/Wash buffer (eBioscience) and stained with α-IgA (Catalog no. 12-4204-82; eBioscience) or α-CD79a. For the staining of intracellular IL-10, cells were restimulated *ex vivo* for 5 h with Cell Stimulation Cocktail plus protein transport inhibitors (eBioscience) and then subjected to cell surface and intracellular FACS staining. Flow cytometric measurements were performed on a FACSVerse flow cytometer (BD Biosciences). Data were analyzed with FlowJo software.

### Immunoblot and immunoprecipitation

The cells were collected and lysed on ice for 30 minutes with RIPA lysis buffer (Beyotime, P0013B) containing PMSF and protease inhibitor (Beyotime, ST505). The lysates were centrifuged, supernatants were collected, mixed with 6 × SDS loading buffer (Beyontime, P0015) and boiled at 95 °C for 10 min. Proteins were electrophoresed at 60 V for 30 min and followed by 90 V for 1 h, then transferred to a PVDF membrane (0.45 μm, Millipore) at 250 mA for 2 h. After blocked with 1% milk at room temperature for 1 h, the membranes were incubated with primary antibodies overnight at 4 °C. After incubating with HRP-conjugated secondary α-mouse or rabbit antibodies, proteins were visualized using chemiluminescence reagent (Millipore). For non-reducing immunoblot, samples were mixed with loading buffer without reducing agent, boiled at 95 °C for 1 min and separated by 7.5% SDS/PAGE at 60 V for 3.5 h.

For all experiments, the same cell lysates were used for both reducing and non-reducing immunoblot analyses, with β-ACTIN probed under reducing conditions as a loading control. For non-reducing immunoblots, the same lysates were applied; however, due to differences in antibody sensitivity, protein loading was adjusted accordingly. Specifically, more protein was loaded for J chain detection compared with IgA detection. For culture supernatant samples, the same volume of each sample was loaded for comparative analysis. For serum samples, those from J chain-deficient mice were loaded at higher dilutions to compensate for elevated IgA levels.

For immunoprecipitation, cells were lysed with NP-40 lysis buffer (Beyotime) containing a protease inhibitor mixture. Precleared lysates were incubated with goat α-mouse IgA (SouthernBiotech) or goat normal IgG (Santa Cruz Biotechnology) overnight at 4 °C with mild rotation, and then Dynabeads Protein G (Invitrogen) were added and rotated for 3 h at 4 °C. Following extensive washing with NP-40 lysis buffer, bound proteins were eluted with 0.1 M Glycine-HCl (pH 2.7), immediately neutralized with 1 M Tris-HCl (pH 8.5), and analyzed by SDS-PAGE under non-reducing conditions and subsequent immunoblotting.

The following Abs were used for immunoblot: HRP-α-mouse-IgA (SouthernBiotech), HRP-α-mouse-lambda (SouthernBiotech), α-J chain (Catalog no. 13688-1-AP; Proteintech), α-β-ACTIN (Catalog no. A1978; Sigma-Aldrich). Polyclonal rabbit Ab against MZB1 were obtained by immunizing rabbits with a mixture of two peptides, LAKAEAKSHTPDASG and SAPTLDDEEKYS, corresponding to amino acids 63–77 and 29–40 of mouse MZB1, respectively.

### Induction and assessment of DSS-induced colitis

The mice were treated with 2.5% Dextran Sodium Sulfate (MP Biomedicals) in the drinking water for 7 days to induce colitis, followed by a 5-day recovery period with regular water. The health condition was monitored daily for 12 days. Disease activity index (DAI) including body weight loss, stool consistency, and stool bleeding was scored as described (30). The colon was collected for length measurement and appearance when the mice were sacrificed at day 12. The colon tissues were then cleaned and fixed in 4% paraformaldehyde for H&E staining. Fecal samples were collected at day 0 and day 4 during experiments and were further detected for IgA by ELISA and immunoblot. The feces at day 0 and day 4 were sent to Sangon Biotech for sequencing the V4 region of the 16S rRNA gene. The results were analyzed as described (31). Pro- and anti-inflammatory cells were analyzed at the end of the experiments.

### Tissue histopathology

The histological assessment of colon injury was performed as described previously by Fu et al. with slight modifications (32). Colonic tissues were removed 12 days after DSS induction and fixed in 4% paraformaldehyde. Paraffin-embedded sections (5 µm) from the distal colon were cut and stained with hematoxylin and eosin (H&E). The evaluation was primarily based on the infiltration of inflammatory cells and epithelial damage, with scoring from 0 to 4 as follows: 0, normal tissue; 1, inflammation with scattered infiltrating inflammatory cells and no signs of epithelial degeneration; 2, multiple foci and/or mild epithelial ulcerations; 3, severe inflammation with marked wall thickening and/or ulcerations in >30% of the tissue section; and 4, inflammation with transmural inflammatory cell infiltration and loss of the entire crypt and epithelium. The assessment was performed in a blinded manner on average fields of view per colon for each mouse.

### Isolation of colonic lamina propria cells

cLP preparations were performed as previously described (33). Single cell suspensions were prepared from colon by a combination of mechanical dissociation and enzymatic degradation. Briefly, colon tissues were cleared of contents and removed of fat tissues. The clean colons were then cut into small pieces and treated for 20 min with predigestion solution (1 mM EDTA, 1 mM DTT, 2% FBS in HBSS w/o Ca^++^ and Mg^++^) at 37 °C under shaking. The tissues were washed and further digested in RPMI 1640 medium containing 2% FBS, 1 mg/ml collagenase IV (Sigma), 0.5 mg/ml dispase II (Roche) and 0.5 mg/ml of DNase I (Sigma) for 1 h at 37 °C under shaking. Samples were then filtered and collected by centrifugation. Lymphocytes were enriched by 40%/70% discontinuous Percoll gradient (GE Healthcare) at 2,000 rpm for 30 min. Cells from the middle layer of the Percoll gradient were collected and washed for further applications.

### ELISA

ELISA was performed as described (28). Briefly, 96-well plates were coated with 5 μg/mL anti-Ig (H+L) (SouthernBiotech), and blocked with PBS containing 1% BSA for 1 h. Diluted samples were then added and incubated for 1 h at room temperature. After washing, HRP-conjugated goat α-mouse IgA (SouthernBiotech) were added and developed with 2,2′-azinobis (3-ethylbenzothiazoline)-6-sulfonic acid solution. For measuring fecal IgA, fecal pellets were collected, weighted and freeze-dried prior to extraction. The dried pellets were then dissolved in sterile PBS containing a protease inhibitor mixture (Sigma-Aldrich). Pellets were vortexed on max speed at 4 °C for 10 min. Debris was removed by centrifugation at 12,000 rpm for 10 min, and the fecal water supernatant was collected. Fecal IgA was then measured by a Mouse IgA ELISA Quantitation Set (Bethyl Laboratories).

### Flow cytometry analysis of IgA^+^ fecal bacteria

The analysis was performed as previously described (34). Briefly, fresh mouse fecal pellets were incubated in PBS (1ml PBS per 10 mg fecal material) for 1 h on ice, homogenized using a vortex mixer and centrifuged (50 *× g*, 15min, 4 °C) to remove large particles. Fecal bacteria in the supernatants were collected, filtered through a 70 μm cell strainer and centrifuged for 5 min (8,000 *× g*, 4 °C). Bacterial pellets were then washed and resuspended in 100 μl blocking buffer (staining buffer containing 10% Normal Rat Serum), incubated for 20 min on ice, and then stained with 100 μl staining buffer (PBS containing 1% BSA) containing PE-conjugated anti-mouse IgA (1:12.5, eBioscience, clone mA-6E1) for 30 min on ice. An PE-conjugated Rat IgG_1_, kappa isotype control was included to identify the stained population. Samples were then washed 3 times and filtered again before flow cytometric analysis.

### Statistical analysis

**Figure 1:**
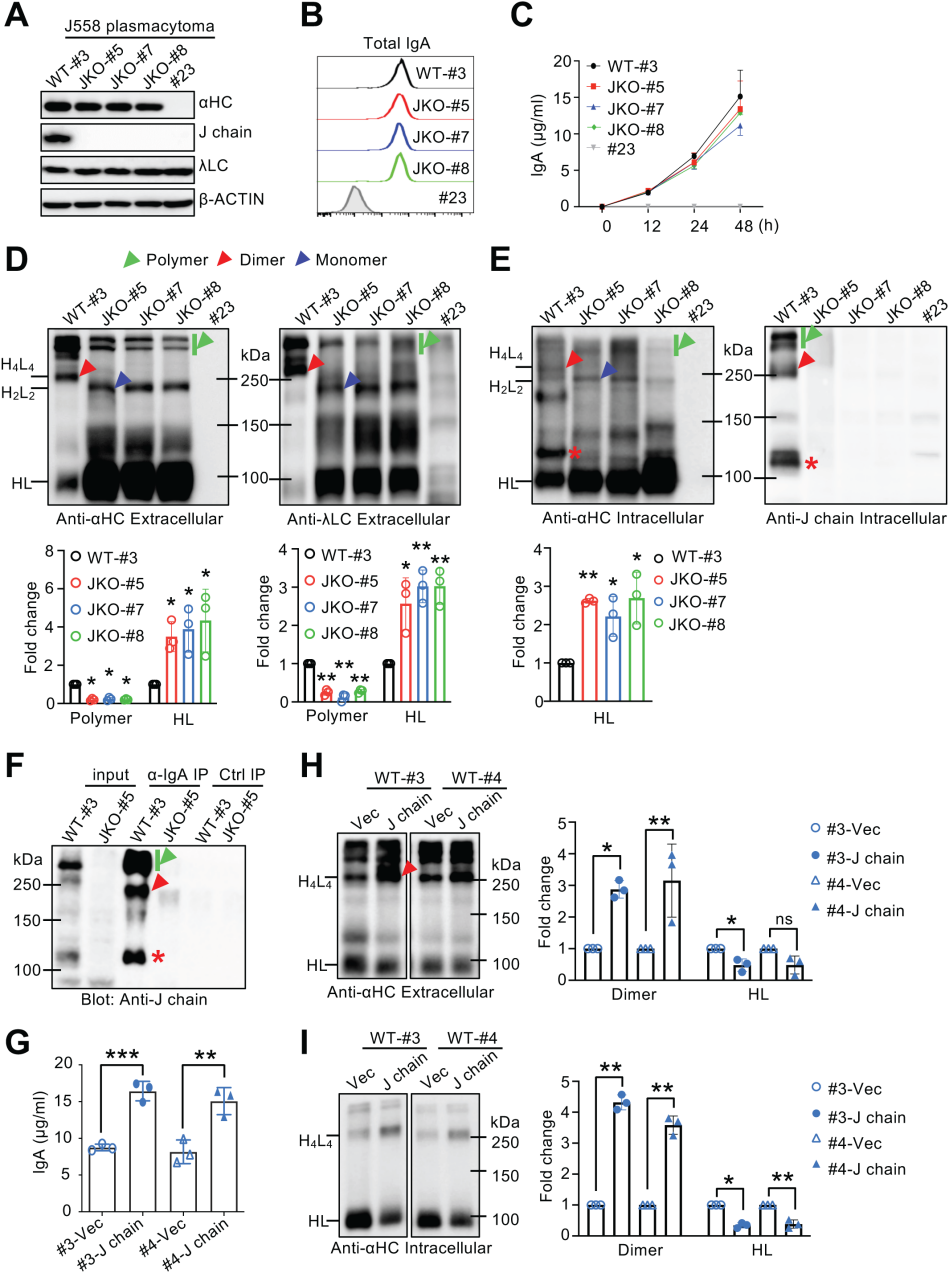
J chain deficiency and overexpression reveal its essential role in IgA polymerization. **(A)** Generation of J chain-deficient J558 cells. Immunoblot analysis for αHC, J chain, λLC and β-ACTIN expression in J558 clones. WT clone #3 served as a positive control, clones #5, #7, and #8 were J chain-deficient, and clone #23, which lost αHC expression, served as a negative control. **(B)** Intracellular staining of total IgA in J558 clones. **(C)** IgA levels in culture supernatants of J558 clones measured by ELISA at the indicated time points. **(D, E)** Non-reducing immunoblot analysis of extracellular **(D)** and intracellular **(E)** IgA in J558 clones. Blots were probed with α-IgA, α-λ light chain, and α-J chain antibodies. Supernatants and cell lysates were collected at 24 h, as indicated in **(C)**. Densitometric quantification of polymeric IgA and HL complexes is shown below panels **(D)** and **(E)**, respectively. **(F)** J chain interacts with HL complexes. J558 WT clone #3 and J chain-deficient clone #5 were lysed and immunoprecipitated with α-IgA Ab. Normal goat IgG was used as negative control (Ctrl) and whole-cell lysate was used as input; non-reducing immunoblot was probed with α-J chain Ab. **(G–I)** Overexpression of J chain in WT J558 cells. Clones #3 and #4 were transduced with retrovirus expressing J chain-IRES-hCD8α or hCD8α alone. **(G)** IgA levels in culture supernatants 48 h post-transduction measured by ELISA. **(H, I)** Non-reducing immunoblot analysis of IgA in culture supernatants **(H)** and cell lysates **(I)**. Densitometric quantification of dimeric IgA and HL complexes is shown to the right of the respective panels. JKO, J chain knockout. Red and blue arrowheads indicate dimeric and monomeric IgA respectively. Green arrowheads indicate polymeric IgA. Red asterisk indicates J chain-containing HL complexes. The same lysates were used for reducing and non-reducing immunoblots in panels **(A, E)**, as well as in panel **(I)** and **Supplementary Figure 1F**. For culture supernatant samples, the same volume of each sample was loaded for comparative analysis. Densitometric quantification of the indicated IgA species was performed from three independent experiments and expressed as fold change relative to controls (set to 1). Means ± SD are shown. “ns, not significant. *P < 0.05; **P < 0.01; ***P < 0.001 (two-tailed unpaired Student’s *t* test).

**Figure 2:**
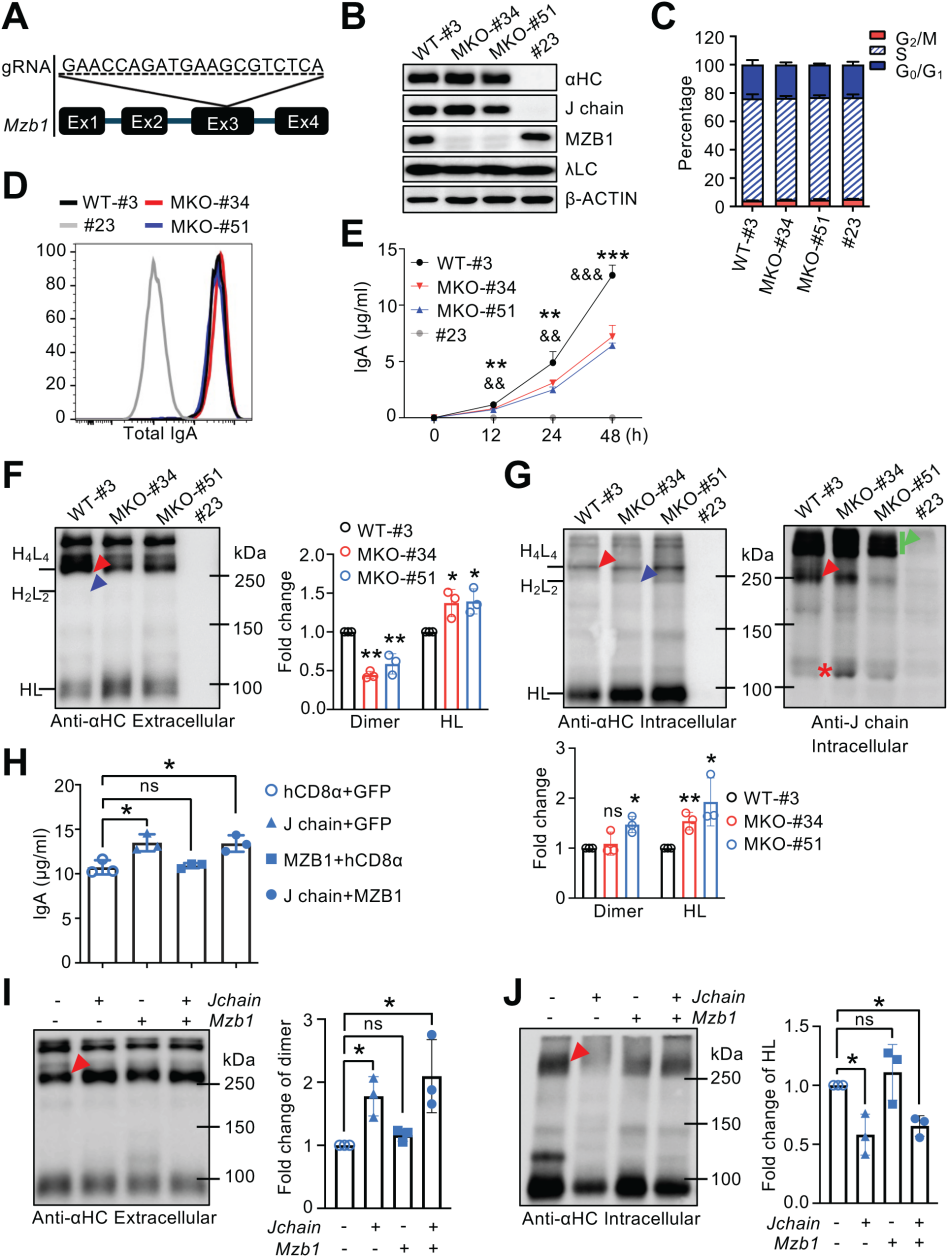
MZB1 deficiency reduces IgA secretion and impairs dimer formation in J558 cells. **(A, B)** Generation of MZB1-deficient J558 cells. **(A)** Position and sequence of the guide RNA targeting the *Mzb1* gene. **(B)** Immunoblot analysis of αHC, J chain, MZB1, λLC and β-ACTIN expression in J558 clones. Clone #3 served as a wild-type control, clones #34 and #51 were MZB1-deficient, and clone #23, which lost αHC expression, served as a negative control. **(C)** Cell cycle analysis of J558 clones. **(D)** Intracellular staining of total IgA in J558 clones. **(E)** IgA levels in culture supernatants of J558 clones measured by ELISA at the indicated time points. **(F, G)** Non-reducing immunoblot analysis of extracellular **(F)** and intracellular **(G)** IgA in J558 clones. Blots were probed with α-IgA and α-J chain antibodies. Supernatants and cell lysates were collected at 24 h, as indicated in **(E)**. Densitometric quantification of dimeric IgA and HL complexes is shown to the right of panel **(F)** and below panel **(G)**, respectively. **(H–J)** Overexpression of MZB1 and/or J chain in WT J558 cells. **(H)** IgA levels in culture supernatants 72 h post-transduction measured by ELISA. **(I, J)** Non-reducing immunoblot analysis of IgA in culture supernatants **(I)** and cell lysates **(J)**. Densitometric quantification of dimeric IgA or HL complexes is shown to the right of the respective panels. MKO, MZB1 knockout. Arrowheads are defined as in **Figure 1**. The same lysates were used for reducing and non-reducing immunoblots. Densitometric quantification of the indicated IgA species was performed from three independent experiments and expressed as fold change relative to controls (set to 1). Means ± SD are shown. ns, not significant. *P < 0.05; **P < 0.01; ***P < 0.001 (two-tailed unpaired Student’s *t* test). * Denotes clone #34 and & denotes clone #51 in **(E)**.

**Figure 3:**
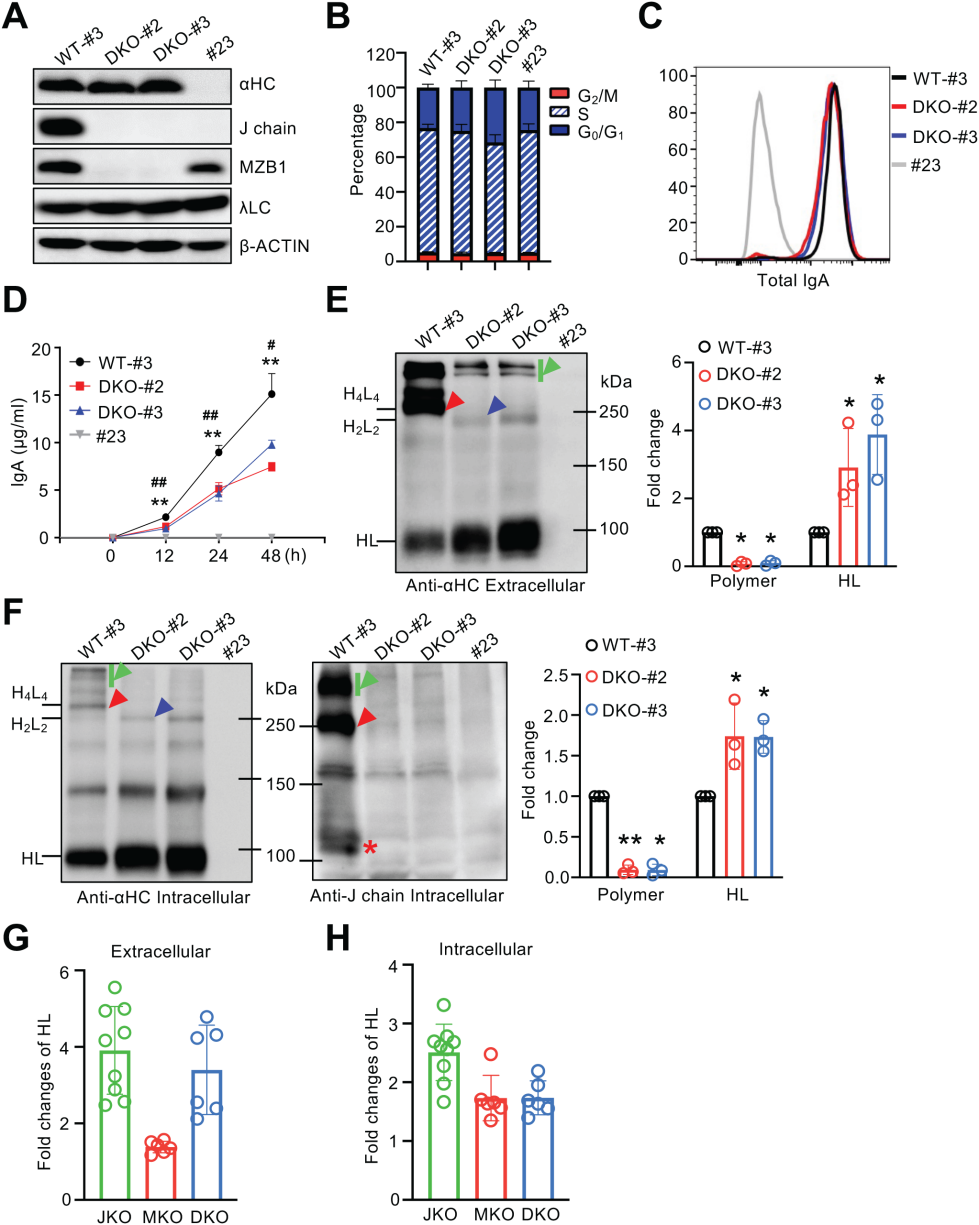
Doubly deficient J558 cells display a combined phenotype of J chain and MZB1 deficiencies. **(A)** Generation of J chain- and MZB1- doubly deficient J558 cells. Immunoblot analysis of αHC, J chain, MZB1, λLC and β-ACTIN expression in J558 clones. Clone #3 served as a wild-type control, clones #2 and #3 were J chain- and MZB1- doubly deficient, and clone #23, which lost αHC expression, served as a negative control. **(B)** Cell cycle analysis of J558 clones. **(C)** Intracellular staining of total IgA in J558 clones. **(D)** IgA levels in culture supernatants of J558 clones measured by ELISA at the indicated time points. **(E, F)** Non-reducing immunoblot analysis of extracellular **(E)** and intracellular **(F)** IgA in J558 clones. Blots were probed with α-IgA and α-J chain antibodies. Supernatants and cell lysates were collected at 24 h, as indicated in **(D)**. Densitometric quantification of polymeric IgA and HL complexes is shown to the right of the respective panels. **(G, H)** Relative ratios of extracellular **(G)** and intracellular **(H)** HL complex band intensities in knockout clones compared with wild-type controls, quantified across **Figures 1**-**3**. Quantification was performed using data from three independent experiments. JKO, J chain knockout; MKO, MZB1 knockout; DKO, double knockout. Arrowheads are defined as in **Figure 1**. The same lysates were used for reducing and non-reducing immunoblots. Densitometric quantification of the indicated IgA species was performed from three independent experiments and expressed as fold change relative to controls (set to 1). Means ± SD are shown. *P < 0.05; **P < 0.01 (two-tailed unpaired Student’s *t* test). * Denotes clone #2 and # denotes clone #3 in **(D)**.

Statistical analyses were performed using GraphPad Prism 10. Unless otherwise indicated, data are presented as means ± SD. Comparisons between two groups were performed using two-tailed unpaired Student’s *t* tests. For fold-change analyses shown in **Figures 1**–**3**, statistical analyses were performed on the original protein density values prior to normalization, before setting control values to 1. Probability values < 0.05 were considered significant; ns, not significant; *P < 0.05; **P < 0.01; ***P < 0.001 or ****P < 0.0001. Data shown are representative of at least two independent experiments unless otherwise noted.

## Results

### J chain is essential for IgA polymerization in J558 cells.

To investigate the role of J chain in IgA polymerization and secretion, we used the mouse plasmacytoma cell line J558, which naturally produces λLC-containing IgA and predominantly secretes polymeric forms (35), making it a suitable model for studying IgA polymerization. The *Jchain* gene was disrupted in J558 cells using CRISPR/Cas9-mediated genome editing with a guide RNA targeting exon 2 (**Supplementary Figure 1A**), generating three independent J chain-deficient clones (JKO-#5, JKO-#7, and JKO-#8). A wild-type (WT) control clone (#3) and an αHC-negative clone (#23) (**Figure 1A**) were included for comparison. We confirmed that cell cycle distribution was comparable among these clones (**Supplementary Figure 1B**). In addition, total IgA expression, as determined by intracellular FACS staining, was comparable among clones (**Figure 1B**), consistent with reducing immunoblots showing no significant differences in total heavy and light chains (**Figure 1A**). Cells were cultured under identical conditions for 48 h, and both supernatants and cell lysates were collected at different time points for analysis. ELISA showed that the overall concentrations of total IgA species in culture supernatants were comparable between J chain-deficient clones and the WT control (**Figure 1C**). However, because ELISA likely detects both assembled and unassembled IgA species without distinguishing between them, these measurements may not accurately reflect the amount of fully assembled IgA. To further assess the distribution of distinct IgA assembly states, we therefore performed immunoblotting under non-reducing conditions. A commercial IgA control was used to assign the dimeric IgA band, and serum from J chain-deficient mice, which predominantly contains monomeric IgA, served as a reference for monomeric migration. Non-reducing immunoblotting revealed a near complete loss of dimeric IgA and a marked reduction of higher-order polymers in J chain-deficient clones, accompanied by prominent accumulation of monomeric IgA (**Figure 1D**). Notably, HL complexes, identified by their reactivity with both anti-HC and anti-LC antibodies, also accumulated in the absence of J chain (**Figure 1D**), suggesting that J chain deficiency impairs not only dimeric IgA formation but also the proper assembly of HL complexes into monomeric IgA (H_2_L_2_). However, we cannot exclude the possibility that the band assigned as the HL complex may also contain HC-only species and/or degradation products. As expected, J chain was detectable in polymeric IgA from WT clone #3 but absent from J chain-deficient clones (**Figure 1E**, right panel; **Supplementary Figure 1C**). Analysis of cell lysates further confirmed these defects, showing impaired dimerization and a concomitant accumulation of HL complexes in J chain-deficient clones (**Figure 1E**, left panel). Of note, in addition to polymers, J chain was also present in HL complexes in WT cells (**Figure 1E**, HL+J marked by *), suggesting that it associates with these intermediates to facilitate their assembly into polymers. Together, these results demonstrate that J chain is essential for assembling HL complexes into dIgA for secretion. Although the levels of total IgA species measured by ELISA appeared unaffected by J chain deficiency (**Figure 1C**), the majority of the secreted IgA consisted of unassembled HL complexes rather than properly formed antibodies. Thus, J chain deficiency did not alter the overall quantity but profoundly impaired the quality of the secreted IgA.

### J chain physically associates with HL complexes and promotes their assembly into polymeric IgA

To determine whether the J chain associates with HL complexes, IgA was immunoprecipitated from WT J558 clone #3 and J chain-deficient clone #5, followed by non-reducing immunoblotting (**Figure 1F**; **Supplementary Figure 1D**). As expected, J chain was detected in dimeric and polymeric IgA fractions. Notably, J chain was also co-precipitated with HL complexes (**Figure 1F**, marked by *), confirming that it directly associates with HL complexes.

To test whether enforced J chain expression enhances IgA polymerization, WT J558 clones #3 and #4 were transduced with retroviruses expressing either an empty vector (pMX-IRES-human CD8α) or J chain (pMX-J chain-IRES-human CD8α). Flow cytometry confirmed >99% transduction efficiency (**Supplementary Figure 1E**), and immunoblotting verified J chain overexpression (**Supplementary Figure 1F**). ELISA showed that culture supernatants from J chain-overexpressing cells contained significantly higher IgA levels than controls (**Figure 1G**). Immunoblot analysis revealed that this increase reflected enhanced polymerization, most prominently of dimeric IgA (**Figures 1H–I**; **Supplementary Figure 1G**). Consistently, HL complexes were reduced in both supernatants (**Figure 1H**) and cell lysates (**Figure 1I**). Thus, enforced J chain expression augmented IgA polymerization and secretion while reducing unassembled intermediates, in contrast to J chain deficiency, which caused massive accumulation of HL complexes and a severe block in polymerization. Collectively, these data demonstrate that the J chain physically associates with HL complexes and promotes their assembly into polymeric IgA for efficient secretion.

### IgA secretion is markedly reduced in MZB1-deficient J558 cells

Having defined the role of J chain, we next investigated how MZB1 contributes to IgA assembly and secretion. The *Mzb1* gene was disrupted using CRISPR/Cas9 with a guide RNA targeting exon 3 (**Figure 2A**), generating two independent MZB1-deficient clones (MKO-#34 and MKO-#51) (**Figure 2B**). Cell cycle distribution and total IgA expression were similar to WT controls (**Figures 2C, D**).

Cells were seeded at equal density and cultured for 48 h, after which supernatants and cell lysates were analyzed. ELISA revealed that IgA secretion was significantly reduced in MZB1-deficient J558 clones compared with WT cells (**Figure 2E**). Non-reducing immunoblotting showed significantly decreased levels of dimeric IgA and a slight increase in HL complexes (**Figure 2F**). Therefore, the level of fully assembled IgA is actually more markedly reduced than suggested by the ELISA results. These observations are consistent with our previous findings in Ag8 plasmacytoma cells, where MZB1 stabilized HL complexes and promoted J chain incorporation into IgA (28). Notably, unlike J chain-deficient J558 cells, MZB1 deficiency did not cause massive accumulation of HL complexes in either the cytoplasm or culture supernatants, likely reflecting reduced stability of HL complexes in the absence of MZB1 (**Figures 2F, G**). MZB1 deficiency also resulted in a modest accumulation of cytoplasmic dimeric IgA in clone #51 (**Figure 2G**, left panel), consistent with reduced dIgA secretion (**Figure 2F**). The impaired secretion of dIgA in clone #51 was associated with reduced J chain incorporation (**Figure 2G**, right panel). Taken together with our previous findings, these results indicate that MZB1 stabilizes HL complexes and facilitates J chain incorporation, thereby promoting efficient IgA polymerization and secretion.

### MZB1 and J chain sequentially bind HL complexes to promote IgA polymerization

To further elucidate the relationship between MZB1 and J chain in IgA assembly, we performed overexpression and double knockout experiments. J558 WT clone #3 was transduced with retroviruses expressing MZB1-IRES-GFP and/or J chain-IRES-human CD8α (**Supplementary Figure 2A**). Transduction efficiency and elevated protein expression were confirmed by flow cytometry and immunoblotting (**Supplementary Figures 2B, C**). ELISA revealed that overexpression of J chain, but not MZB1, enhanced IgA secretion (**Figure 2H**). Consistent with the results shown in **Figure 1**, only J chain overexpression increased the secretion of dimeric IgA (**Figures 2H, I**), accompanied by a reduction of cytoplasmic HL complexes (**Figure 2J**). These data suggest that J chain is the limiting factor for polymerization, whereas endogenous MZB1 is sufficient to stabilize HL complexes.

To examine their cooperative roles, we next generated J558 cells doubly deficient in MZB1 and J chain (DKO-#2 and DKO-#3) using CRISPR/Cas9 (**Figure 3A**). As with single knockouts, cell cycle distribution and total IgA expression were comparable to those of WT controls (**Figures 3B, C**). Under identical culture conditions, double-deficient J558 clones secreted significantly less IgA (**Figure 3D**), closely resembling the phenotype of MZB1 deficiency (**Figure 2E**). Analysis of IgA assembly revealed combined features of both J chain and MZB1 deficiencies, including markedly impaired polymerization, loss of detectable dimers, and accumulation of HL complexes (**Figures 3E, F**, left panel). Intracellular J chain detection confirmed that the accumulation of HL complexes was associated with the absence of J chain, whereas HL+J complexes were readily detected in WT cells (**Figure 3F**, right panel). To quantify these effects, we calculated the relative HL complex band intensities in knockout clones (**Figures 1**-**3**) compared with WT controls. As shown in **Figures 3G, H**, J chain deficiency caused the greatest accumulation of HL complexes, reflecting a profound blockade in assembly. Double deficiency ranked second, as the additional loss of MZB1 caused partial degradation of the HL pool in the context of J chain deficiency. MZB1 single deficiency resulted in the least accumulation of HL complexes, indicating reduced HL stability while still permitting some HL accumulation due to impaired J chain-driven assembly. Taken together, these results demonstrate that MZB1 and J chain act sequentially to ensure proper IgA assembly and secretion: MZB1 first stabilizes HL complexes and facilitates J chain incorporation, after which J chain drives the assembly of HL complexes into dimers and higher-order polymers.

### MZB1-deficient mice develop severe colitis upon DSS treatment, whereas J chain-deficient mice do not show increased susceptibility

**Figure 4:**
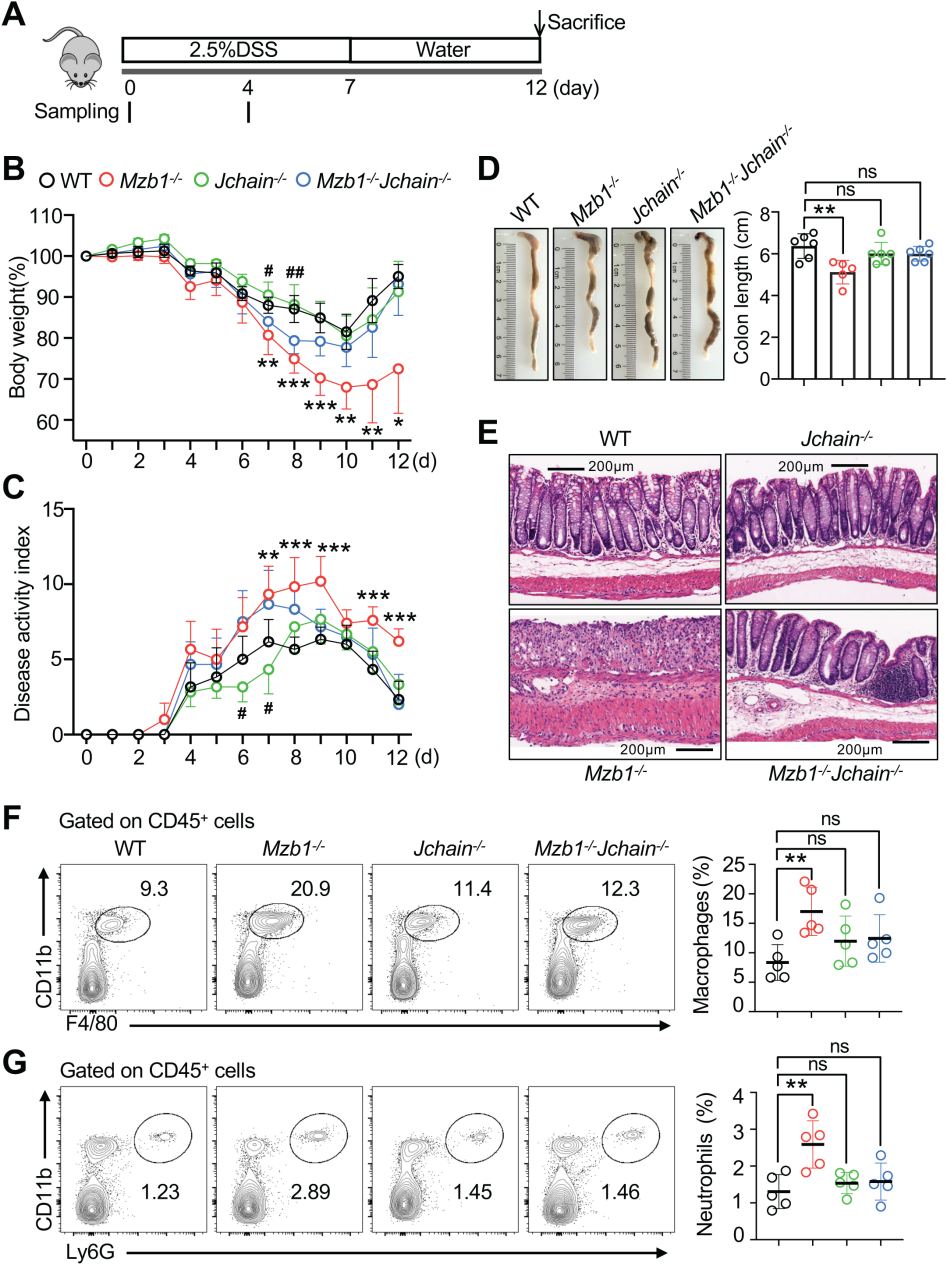
Differential susceptibility to DSS-induced colitis among mouse strains. **(A)** Schematic illustration of the DSS-induced acute colitis protocol. Mice were administered 2.5% DSS in drinking water for 7 days, followed by regular water for 5 days. Serum and fecal samples were collected on day 0 and day 4, as indicated. Mice were monitored daily until sacrifice on day 12. **(B)** Body weight changes after DSS administration. The initial body weight of each mouse was defined as 100%. **(C)** Disease activity index scores. **(D)** Comparison of colon length and representative images of colons at the end of the experiment (day 12). **(E)** Histopathological changes in the distal anorectal region of colon tissues. **(F, G)** Frequencies of macrophages (CD45^+^CD11b^+^F4/80^+^) and neutrophils (CD45^+^CD11b^+^Ly6G^+^) in the colonic lamina propria. Five to six male mice per group were analyzed. Each symbol represents an individual mouse; lines indicate means ± SD. ns, not significant. *p < 0.05; **p < 0.01; ***p < 0.001 (two-tailed unpaired Student’s *t* test). * Denotes *Mzb1^-/-^* mice and # denotes *Mzb1^-/-^Jchain^-/-^* mice in **(B, C)**. Data are representative of at least three independent experiments.

Through gene knockout and overexpression experiments in J558 plasmacytoma cells, we clarified the roles of J chain and MZB1 in IgA assembly and polymerization. We next explored their physiological relevance by assessing how altered IgA quantity and quality affect susceptibility to intestinal inflammation. To this end, we generated *Jchain^-/-^*, *Mzb1^-/-^* and double-deficient *Mzb1^-/-^Jchain^-/-^* mice. These animals were subjected to DSS-induced colitis by administration of 2.5% DSS in drinking water for seven days, followed by five days of normal water (**Figure 4A**). *Mzb1^-/-^* mice exhibited significantly greater body-weight loss compared with WT controls, whereas *Jchain^-/-^* mice showed no significant difference, with only mild weight loss at late time points (**Figure 4B**). Double-deficient mice displayed an intermediate phenotype. Disease activity index (DAI) scores followed the same trend (**Figure 4C**). Upon sacrifice, *Mzb1^-/-^* mice had the shortest colons (**Figure 4D**) and displayed the most severe histopathology, with extensive loss of epithelial integrity and massive infiltration of inflammatory cells (**Figure 4E**). In contrast, histological changes in J chain-deficient and double-deficient mice were not significantly different from those observed in WT controls, consistent with the colon length measurements (**Figure 4D**) and the histological scoring analysis (**Supplementary Figure 3A**). Notably, one *Mzb1^-/-^* mouse succumbed to severe colitis by day 9, while all other mice survived.

To examine why *Mzb1^-/-^* and *Jchain^-/-^* mice differ in their susceptibility to DSS, we analyzed inflammatory and regulatory cell populations in colonic tissue and spleen. *Mzb1^-/-^* mice had significantly increased macrophages and neutrophils in the colon compared with WT mice (**Figures 4F, G**), accompanied by similar increases in the spleen (**Supplementary Figures 3B–D**), indicating aggravated systemic inflammation. Double-deficient and *Jchain^-/-^* mice also displayed increased splenic inflammatory cells, particularly macrophages, but to a lesser extent than *Mzb1^-/-^* mice. We next assessed IL-10-producing cells in the lamina propria, a key regulatory population (36, 37). Plasmablasts/plasma cells (PBs/PCs) were identified as CD79a^+^IgA^+^ cells, following a previously described strategy (36) in which CD79a^+^IgA^+^ cells in the colonic lamina propria were shown to largely correspond to PBs/PCs. CD138 was not used to define PBs/PCs since its surface expression can be substantially reduced by enzymatic digestion during lamina propria cell isolation. *Mzb1^-/-^* mice showed a significant reduction in IL-10-producing PBs/PCs compared with WT controls (**Supplementary Figures 4A, B**). Taken together, these results demonstrate that the *Mzb1^-/-^* mice develop severe DSS-induced colitis, associated with increased inflammatory cell infiltration and reduced IL-10-producing regulatory cells, leading to aggravated intestinal and systemic inflammation. In contrast, *Jchain^-/-^* mice do not show increased susceptibility, despite impaired IgA polymerization.

### Both the quantity and quality of IgA contribute to the suppression of DSS-induced colitis

**Figure 5:**
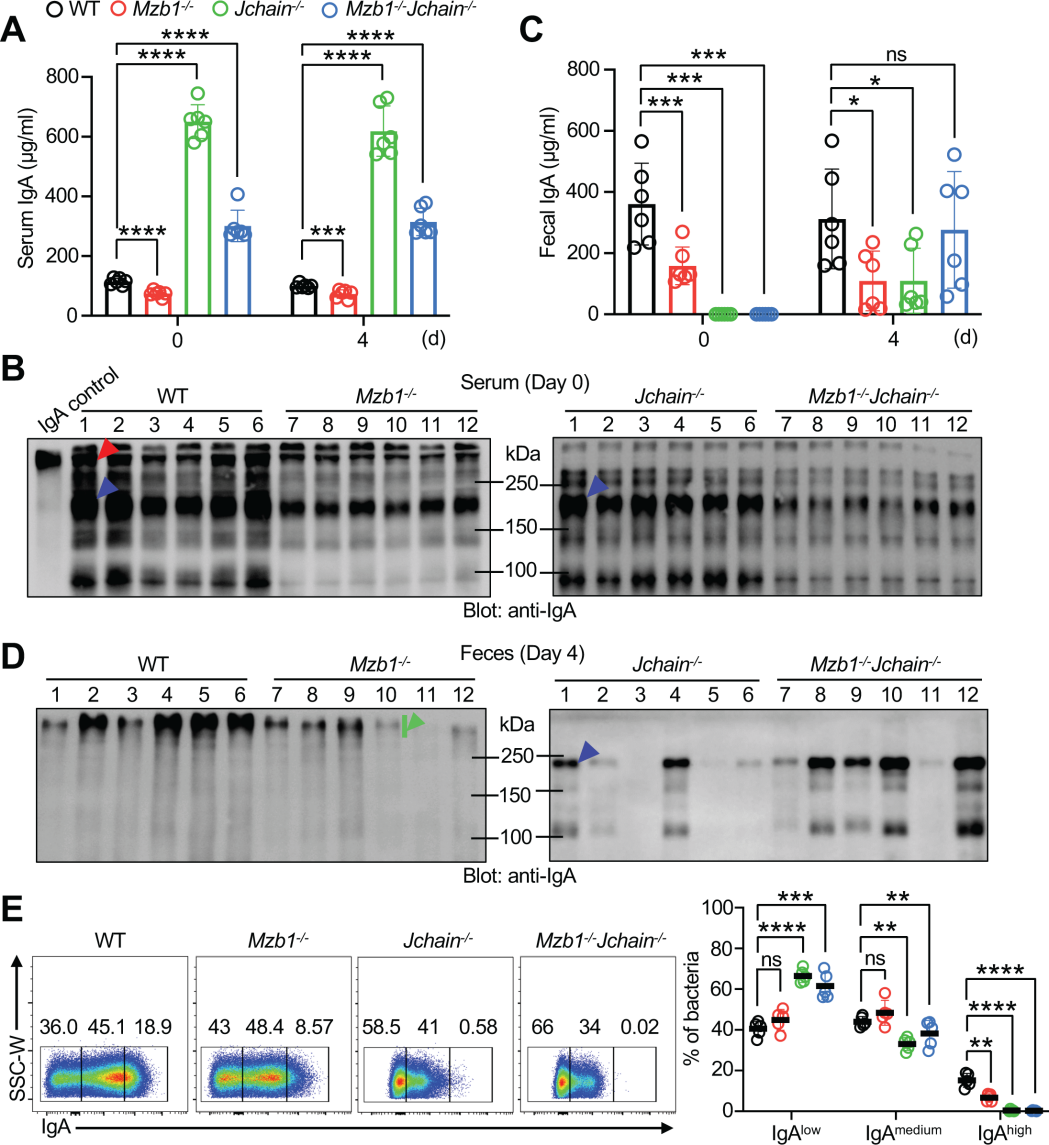
Altered IgA quantity and quality influence susceptibility to DSS-induced colitis. **(A–D)** Assessment of IgA at early time points during model induction. **(A)** Serum IgA levels at days 0 and 4 of DSS treatment, measured by ELISA. **(B)** Non-reducing immunoblot analysis of serum IgA at steady state (day 0) in each mouse group, showing dimeric and monomeric forms. A commercial mouse IgA (Catalog no. 0106-01, SouthernBiotech) was used as a control. **(C)** Fecal IgA levels at days 0 and 4 of DSS treatment, measured by ELISA. **(D)** Non-reducing immunoblot analysis of fecal IgA at day 4. **(E)** Analysis of IgA coating on fecal bacteria from mice at day 4. Frequencies of IgA^low^, IgA^medium^ and IgA^high^ populations are shown (right), with representative FACS plots of IgA staining (left). Arrowheads are defined as in **Figure 1**. Means ± SD of 5–6 mice per group are shown. ns, not significant. *p < 0.05; **p < 0.01; *** p < 0.001; **** p < 0.0001 (two-tailed unpaired Student’s t test). Data are representative of at least three independent experiments.

To investigate the mechanisms underlying the different severities of colitis among mouse strains, we evaluated the quantity and quality of IgA in serum and feces during DSS-induced colitis, focusing on early time points before or during disease onset. In serum (**Figure 5A**), *Mzb1^-/-^* mice exhibited significantly reduced IgA levels both at steady state (day 0) and at day 4, consistent with impaired IgA secretion in the absence of MZB1 and in line with our findings in J558 plasmacytoma cells. By contrast, *Jchain^-/-^* mice displayed markedly elevated serum IgA levels, approximately eightfold higher than WT controls, reflecting the accumulation of monomeric IgA as previously reported (38–40). Using commercial IgA control and WT serum as controls, we found that IgA species detected in *Jchain^-/-^* serum (**Figure 5B**) closely resemble those previously reported in the literature (38), including a predominant monomeric IgA band, several minor bands migrating above the monomer, and a lower molecular weight band below 100-kDa. *Mzb1^-/-^Jchain^-/-^* mice also showed significantly higher serum IgA levels than WT mice, though lower than *Jchain^-/-^* mice owing to decreased HL stability in the absence of MZB1. In feces (**Figure 5C**), *Mzb1^-/-^* mice had lower IgA levels at both day 0 and day 4, consistent with reduced overall IgA output. In contrast, *Jchain^-/-^* and *Mzb1^-/-^Jchain^-/-^* mice had almost no detectable IgA in feces prior to DSS treatment, since polymerization and transport of IgA require the J chain (4, 38). However, by day 4, fecal IgA levels in both strains became comparable to WT mice, although with marked inter-individual variability, likely due to leakage of serum IgA into the gut lumen following DSS-induced epithelial barrier disruption (41). Non-reducing immunoblotting confirmed that this leaked IgA was present as monomers (**Figure 5D**), whereas in *Mzb1^-/-^* mice, fecal IgA remained primarily polymeric but at lower abundance. Notably, despite more pronounced histological damage in *Mzb1^-/-^* mice (**Figure 4E**; **Supplementary Figure 3A**), fecal IgA leakage was not increased in this group. This likely reflects the markedly reduced serum IgA levels in *Mzb1^-/-^* mice, leaving little IgA available to leak into the intestinal lumen. These results demonstrate that impaired IgA production predisposes to severe inflammation, as observed in *Mzb1^-/-^* mice, whereas elevated serum IgA in *Jchain^-/-^* mice provides protection against DSS-induced colitis. This protective effect likely involves multiple mechanisms, including leakage of serum IgA into the gut lumen and systemic anti-inflammatory functions of circulating IgA.

### Different IgA levels and forms are associated with differences in gut microbiota composition

The gut microbiota critically influences DSS-induced colitis, and IgA shapes microbial composition and maintains mucosal homeostasis (42–44). To assess the role of different IgA forms in regulating gut microbial communities, we quantified IgA coating of fecal bacteria on day 4 after DSS treatment by flow cytometry (**Figure 5E**; **Supplementary Figure 4C**). The proportion of bacteria highly coated with IgA was markedly reduced in *Mzb1^-/-^* mice compared with WT controls. In *Jchain^-/-^* and *Mzb1^-/-^Jchain^-/-^* mice, the IgA^high^ bacterial population was almost undetectable, although both strains maintained a substantial IgA^medium^ population. However, the staining intensity of the IgA^medium^ population in the absence of *Jchain* was significantly reduced, consistent with the loss of dimeric IgA. IgA^medium^ staining in *Mzb1^-/-^* mice was also slightly reduced compared with WT mice, reflecting reduced levels of dimeric IgA (**Supplementary Figure 4D**). Overall, the reduction in IgA-coated bacteria suggests less IgA binding per bacterium, possibly reflecting lower avidity and/or reduced polyreactivity of IgA in the absence of MZB1 and J chain. In the case of J chain deficiency, IgA cannot associate with the secretory component, which can bind microbial species through non-canonical interactions. Its absence may therefore further reduce IgA binding to intestinal bacteria.

**Figure 6:**
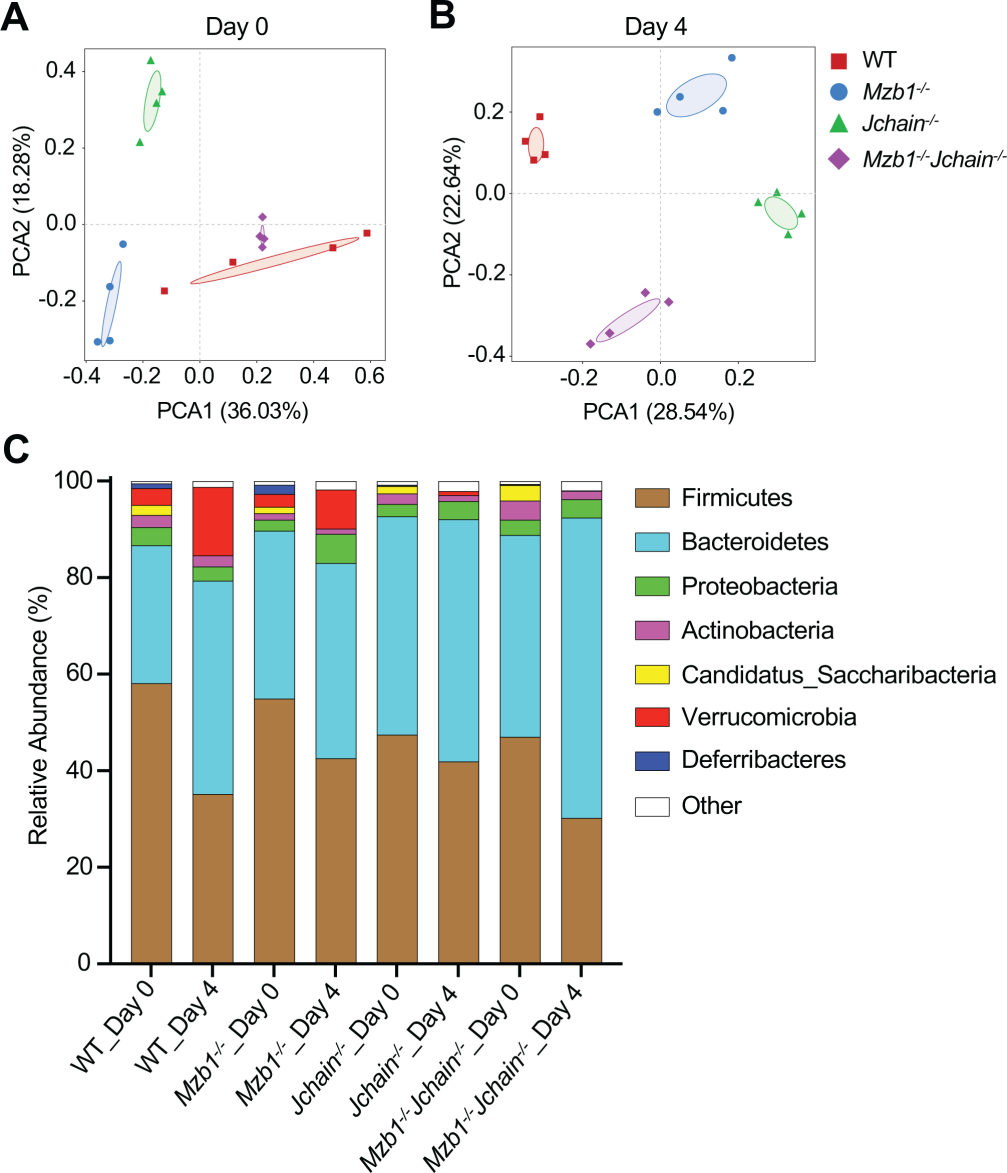
Altered gut microbiota composition during DSS-colitis among mouse strains. **(A, B)** PCA, Principal coordinate analysis showing differences in gut microbial community composition among the indicated mouse strains before DSS treatment (Day 0) **(A)** and after DSS treatment (Day 4) **(B)**. **(C)** Relative abundance of bacterial phyla in fecal samples from each group. Colors correspond to the indicated bacterial phyla; those accounting for <5% of total sequences were combined as “Other”.

To determine whether altered IgA binding affected microbial composition, we performed 16S rRNA gene sequencing of fecal samples collected before and four days after DSS administration. Principal component analysis (PCA) of fecal microbiota at baseline (day 0) revealed a clear genotype-dependent clustering, with *Jchain^-/-^* and *Mzb1^-/-^Jchain^-/-^* mice exhibiting a distinct microbial composition compared to WT and *Mzb1^-/-^* mice (**Figure 6A**). Distinct microbial profiles were also observed among the four genotypes after DSS administration (**Figure 6B**).

At day 0, at the phylum level, the microbial communities of WT mice and *Mzb1^-/-^* mice were quite similar. However, the communities of *Jchain^-/-^* and *Mzb1^-/-^Jchain^-/-^* mice differed significantly from those of WT mice, notably exhibiting near-complete absence of Verrucomicrobia and Deferribacteres (**Figure 6C**). This discrepancy aligns with the critical role of IgA in regulating gut microbiota composition, as evidenced by the absence of IgA in the intestines of *Jchain^-/-^* and *Mzb1^-/-^Jchain^-/-^* mice (**Figure 5C**). Across all genotypes, from day 0 to day 4, there was a decrease in Firmicutes and Candidatus_Saccharibacteria, and an increase in Bacteroidetes, indicating that DSS induces inflammatory dysbiosis (**Figure 6C**). Notably, at day 4 of DSS treatment, *Mzb1^-/-^* mice displayed the most pronounced expansion of Proteobacteria, which reflecting a failure of the epithelial barrier to restrain opportunistic pathobionts (45), and correlated with their severe colitis. In addition, compared with *Jchain^-/-^* and *Mzb1^-/-^Jchain^-/-^* mice, WT animals exhibited a marked expansion of Verrucomicrobia at day 4 (**Figure 6C**). Verrucomicrobia are known to be highly coated with IgA in the healthy human gut and are widely regarded as beneficial commensals (42). Unexpectedly, compared with day 0, *Jchain^-/-^* mice showed the smallest change in overall microbial relative abundance at day 4 (**Figure 6C**), which appears to contrast with the prevailing view that IgA is crucial for maintaining gut microbial homeostasis and diversity, and that its absence typically leads to dysbiosis and an increased propensity for inflammation. However, this limited microbial perturbation is consistent with the relatively mild colitis phenotype observed in *Jchain^-/-^* mice. Moreover, the microbiota composition of the *Mzb1^-/-^Jchain^-/-^* mice was more similar to that of *Jchain^-/-^* mice and markedly different from that of *Mzb1^-/-^* mice, which parallels the fact that both *Mzb1^-/-^Jchain^-/-^* and *Jchain^-/-^* mice develop milder colitis than *Mzb1^-/-^* mice. Together, these data indicate that distinct IgA patterns, defined by differences in quantity and polymeric state, are associated with differences in gut microbial communities during DSS-induced colitis.

## Discussion

Our earlier work established that MZB1 mediates the stability of HL complexes and facilitates the secretion of J chain-containing dimeric IgA (28). In the present study, we further demonstrate that MZB1 stabilizes HL complexes to enable their efficient association with the J chain, which subsequently drives rapid assembly into IgA dimers and higher-order polymers. These findings confirm and extend our previous observations and uncover a previously unrecognized role for the J chain in physically interacting with HL complexes and mediating their assembly into the dimeric and polymeric IgA. Thus, MZB1 and the J chain cooperatively regulate the quantity and quality of IgA, respectively.

When an Ig HC is newly synthesized, the ER chaperone BiP binds to its CH1 domain, maintaining it in an unfolded state until a LC becomes available. LC binding to the HC via an inter-chain disulfide bond induces CH1 folding and displaces BiP (46). This BiP-CH1-LC cycle acts as a quality-control checkpoint to ensure the proper folding and assembly of the HC-LC complexes. In the case of αL complexes, our previous study showed that MZB1 binds to the αtp in a manner dependent on the penultimate cysteine residue. Intriguingly, both MZB1 and the J chain target the same αtp region (47, 48). Our data suggest a sequential mechanism: MZB1 first binds HL complexes to mask the αtp and prevent misfolding, after which the J chain displaces MZB1 by forming a disulfide bond with the αtp, a scenario analogous to how LC binding displaces BiP from CH1. In the absence of MZB1, HL complexes may misfold before associating with the J chain, leading to their degradation and ultimately reducing the amount of secreted IgA.

Unlike mice, which possess only one IgA isotype, humans have two IgA isotypes (IgA1 and IgA2). Cryo-EM structural analyses of human and mouse secretory IgA reveal that despite sequence heterogeneity, human and mouse SIgA share highly conserved core Fc architectures, and most residues at the interface between the J chain and IgA are conserved (16, 49). Additionally, Benjamin Geary et al. reported that MZB1 is required for optimal IgM and IgA secretion by primary human plasmablasts (50). Based on these findings, we speculate that MZB1 plays a role in human IgA biogenesis analogous to that reported here in mice.

A key finding of the present study is that J chain is not merely a passive late-stage component required for IgA dimer formation but also actively participates in the assembly process. Specifically, the J chain associates with HL complexes through interactions with the αtp, displacing MZB1 and driving dimer formation. The massive accumulation of intracellular HL complexes in the absence of the J chain supports this model. Notably, in J chain-sufficient cells, monomeric IgA was barely detectable in either cell lysates or culture supernatants, suggesting that J chain association rapidly drives HL complexes into dimeric or polymeric forms, leaving monomeric IgA as only a transient intermediate.

Unexpectedly, J chain-deficient J558 cells secreted large amounts of HL complexes into the culture supernatant. Similarly, *Jchain^-/-^* and *Mzb1^-/-^Jchain^-/-^* mice contained similar smaller IgA-related species in serum and feces during colitis. These findings highlight multiple roles of the J chain in IgA biosynthesis and quality control beyond its canonical function in dimer formation: 1) association with HL complexes and assembly of dimeric IgA; 2) suppression of monomeric IgA generation; 3) prevention of secretion of unassembled HL intermediates; and 4) mediation of epithelial transcytosis via pIgR.

Although HL complexes were prominently observed in the present study using a J558-based system, their broader relevance across physiological contexts remains to be established. Previous studies using non-reducing immunoblot analyses of IgA have also revealed species migrating below the band assigned as monomeric IgA (15, 20, 51); however, the absence of molecular weight markers in these reports limits the precise assignment of these bands. Based on their designation and migration patterns, it is possible that HL-like species may have been observed but were not specifically assigned or emphasized. Future studies using additional plasma cell lines, as well as primary mouse and human plasma cells, will be important to determine the generality of these findings. In addition, further biochemical characterization using approaches such as SE-HPLC-MALS would provide additional validation, particularly for broadly migrating bands such as HL complexes, where it remains unclear whether they may also contain HC-only species or degradation products.

Another important question concerns the molecular mechanisms that regulate HL stabilization and IgA assembly. MZB1 has been proposed to function within a broader ER chaperone network that supports immunoglobulin folding and polymerization. Previous work showed that co-expression of tagged human MZB1 in plant cells did not increase dimeric IgA production, possibly because essential interaction partners are absent in this heterologous system (52). Several ER chaperones have been implicated in immunoglobulin assembly, including BiP and GRP94, which participate in early folding steps of Ig heavy chains, as well as ERp44, which promotes J chain incorporation during IgM polymerization (53). In addition, post-translational modifications of MZB1 have recently been shown to regulate optimal IgM and IgA secretion in human plasmablasts (50). Together, these observations suggest that MZB1 may cooperate with additional ER chaperones and regulatory factors to control HL stabilization and J chain incorporation during IgA assembly. Future studies dissecting these interactions will help clarify how ER quality control pathways coordinate the formation of distinct IgA molecular species.

Both the quantity and quality of IgA critically influence intestinal immune protection. As expected, *Mzb1^-/-^* mice, which exhibit reduced secretion of dimeric IgA, showed higher susceptibility to DSS-induced colitis, indicating that sufficient amounts of secretory IgA are essential for shaping microbiota composition and preventing inflammation. In contrast, *Jchain^-/-^* mice did not develop more severe colitis than WT controls. Our data show that by day 4 of DSS treatment when epithelia barrier disruption occurs, monomeric IgA becomes detectable in the gut lumen of *Jchain^-/-^* mice. This monomer IgA may attenuate intestinal inflammation by modulating the microbiota, even though its affinity for bacteria is lower than that of polymeric IgA. Although HL complexes were also detected in the gut at this stage, their functional contribution remains unclear. Future studies could directly test the roles of systemic monomeric IgA or HL complexes by administering defined amounts of purified IgA species during DSS-induced colitis.

It is worth noting that DSS-induced colitis results from epithelia barrier damage, allowing systemic IgA leakage into the gut. Under such conditions, the absence of the J chain does not exacerbate inflammation. However, in models of colitis driven by impaired regulatory pathways, such as IL-10-deficiency where barrier integrity remains intact, *Jchain^-/-^* mice may exhibit increased susceptibility. This possibility is currently under investigation.

In conclusion, our findings define a cooperative mechanism by which MZB1 and the J chain orchestrate IgA biosynthesis and secretion. MZB1 functions as an early chaperon that stabilizes HL complexes and prepares them for J chain incorporation, while the J chain actively drives dimer and polymer formation, prevents secretion of immature intermediates, and enables epithelial transcytosis. Together, these molecules ensure the production of sufficient quantities of high-quality polymeric IgA essential for maintaining mucosal immune homeostasis.

## Data Availability

The datasets presented in this article are not readily available. Any additional data is available from the corresponding author upon request. Requests to access the datasets should be directed to Ji-Yang Wang, wang@fudan.edu.cn.
