# Supplementary material for: Cooperative control of IgA synthesis and secretion by MZB1 and the J chain
Source: Front Immunol. 2026 Apr 17;17:1744147. doi: 10.3389/fimmu.2026.1744147 (PMC13132693; doi:10.3389/fimmu.2026.1744147)
Supplement: Supplementary file 1 [file DataSheet1.pdf]

Supplementary Material

Supplementary Figures

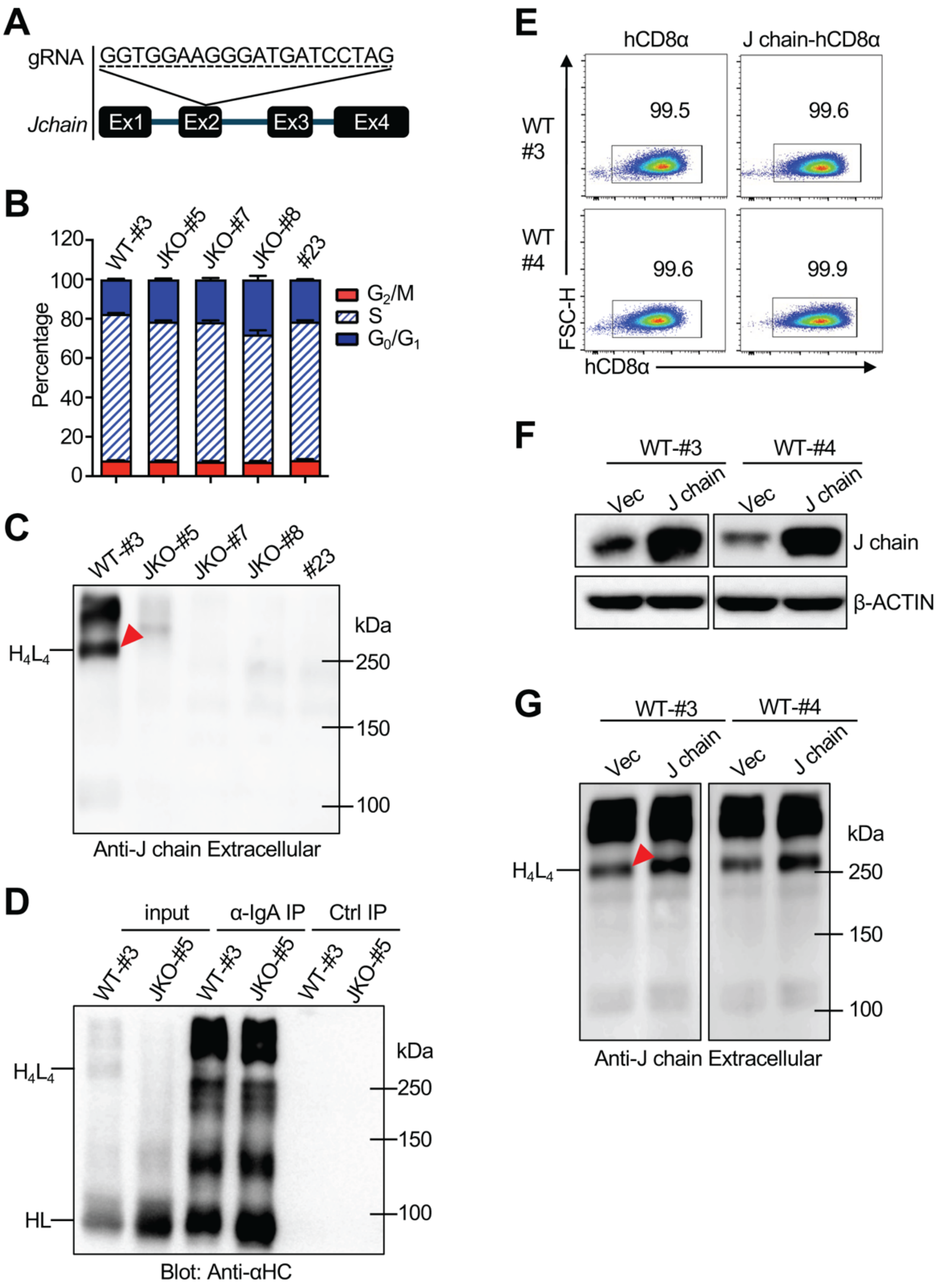

Supplementary Figure 1. J chain deficiency and overexpression.
(**A**) Position and sequence of the guide RNA used for *Jchain* targeting. (**B**) Cell cycle analysis of J558 clones. (**C**) Non-reducing immunoblot analysis of extracellular J chain in J558 clones. The sample sets are the same as those shown in **Figure 1D**. (**D**) The same immunoprecipitates as in **Figure 1F** were analyzed by non-reducing immunoblot and probed with α-IgA Ab. (**E**-**G**) Validation of J chain overexpression in WT J558 cells. Clones #3 and #4 were transduced with retrovirus expressing J chain-IRES-hCD8α or hCD8α alone. (**E**) Transduction efficiency (>99%) measured by flow cytometry. (**F**) Immunoblot confirming J chain overexpression; β-ACTIN was used as a loading control. (**G**) Non-reducing immunoblot analysis of J chain in culture supernatants. The samples are identical to those in **Figure 1H**.

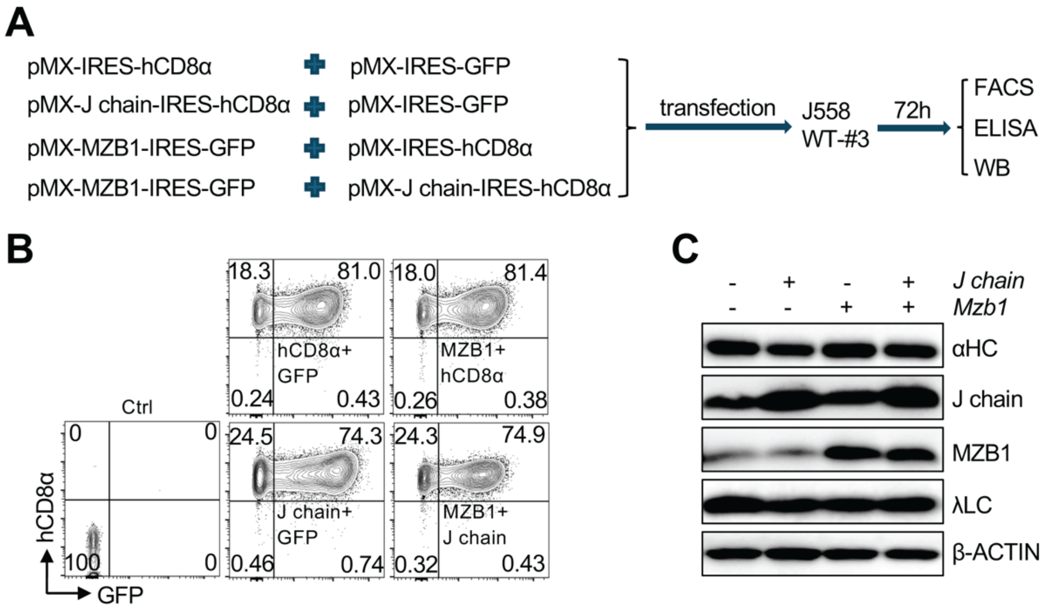

Supplementary Figure 2. Validation of J chain and/or MZB1 overexpression in J558 cells.
(A) Experimental scheme. Clone #3 was transduced with retroviruses expressing: hCD8α and GFP; J chain-IRES-hCD8α and GFP; MZB1-IRES-GFP and hCD8α; or MZB1-IRES-GFP and J chain-IRES-hCD8α. Cells were analyzed 72 h post-transduction by flow cytometry, ELISA and immunoblotting. **(B)** Transduction efficiency measured by flow cytometry. (**C**) Immunoblot confirming J chain and MZB1 overexpression; αHC and λLC were included as additional controls, and β-ACTIN was used as a loading control.

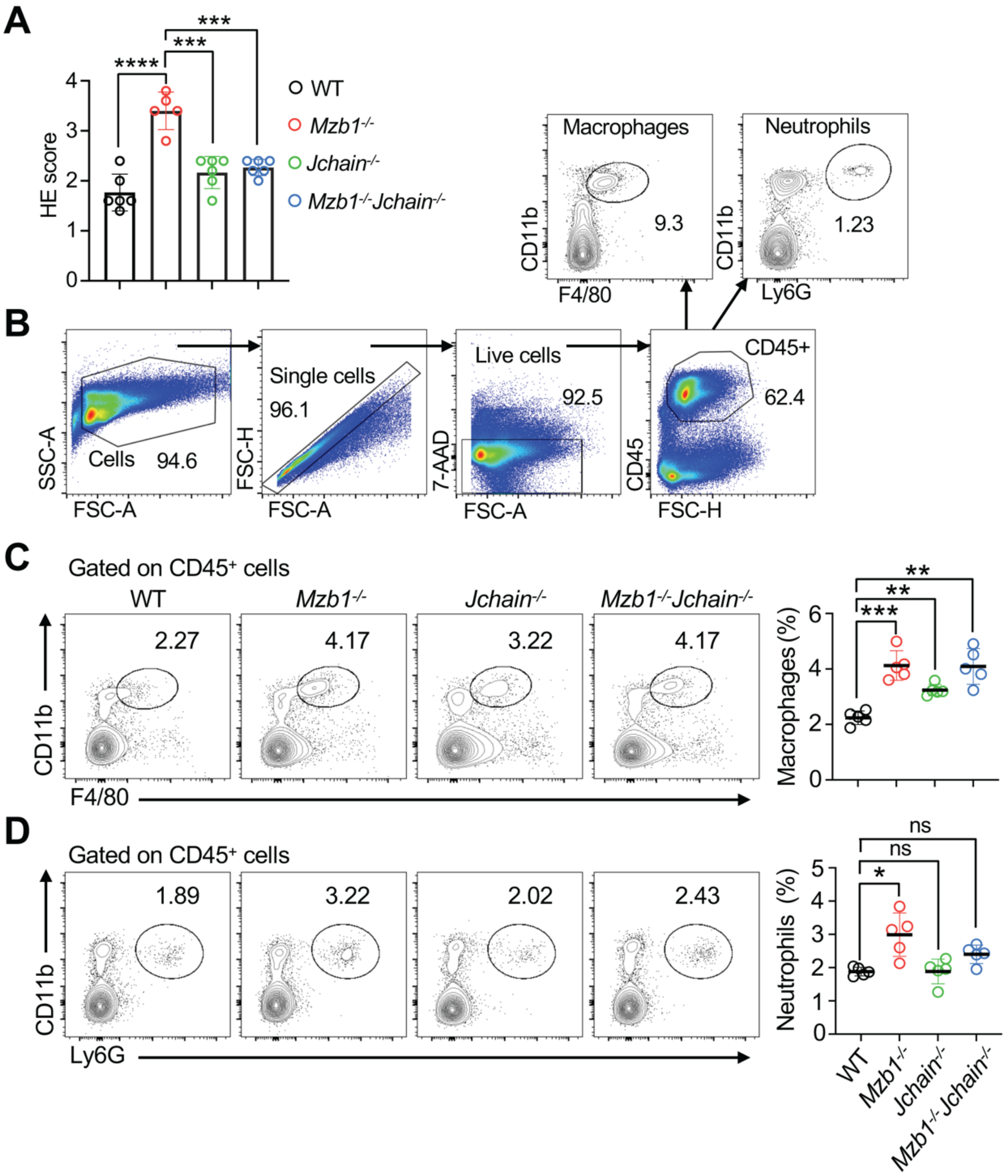

Supplementary Figure 3. Histological scores and myeloid cell composition in mice with DSS-induced colitis.

(**A**) The histological score of **Figure 4E**. (**B**) Representative gating strategies for macrophages ($CD45^+CD11b^+F4/80^+$) and neutrophils ($CD45^+CD11b^+Ly6G^+$) used in **Figure 4F** and **4G** as well as in panels (**C**) and (**D**). (**C**, **D**) Frequencies of macrophages (**C**) and neutrophils (**D**) in the spleen. Means ± SD are shown. ns, not significant. *$P <$ 0.05; **$P <$ 0.01; ***$P <$ 0.001(two-tailed unpaired Student's t test). Data are representative of at least two independent experiments.

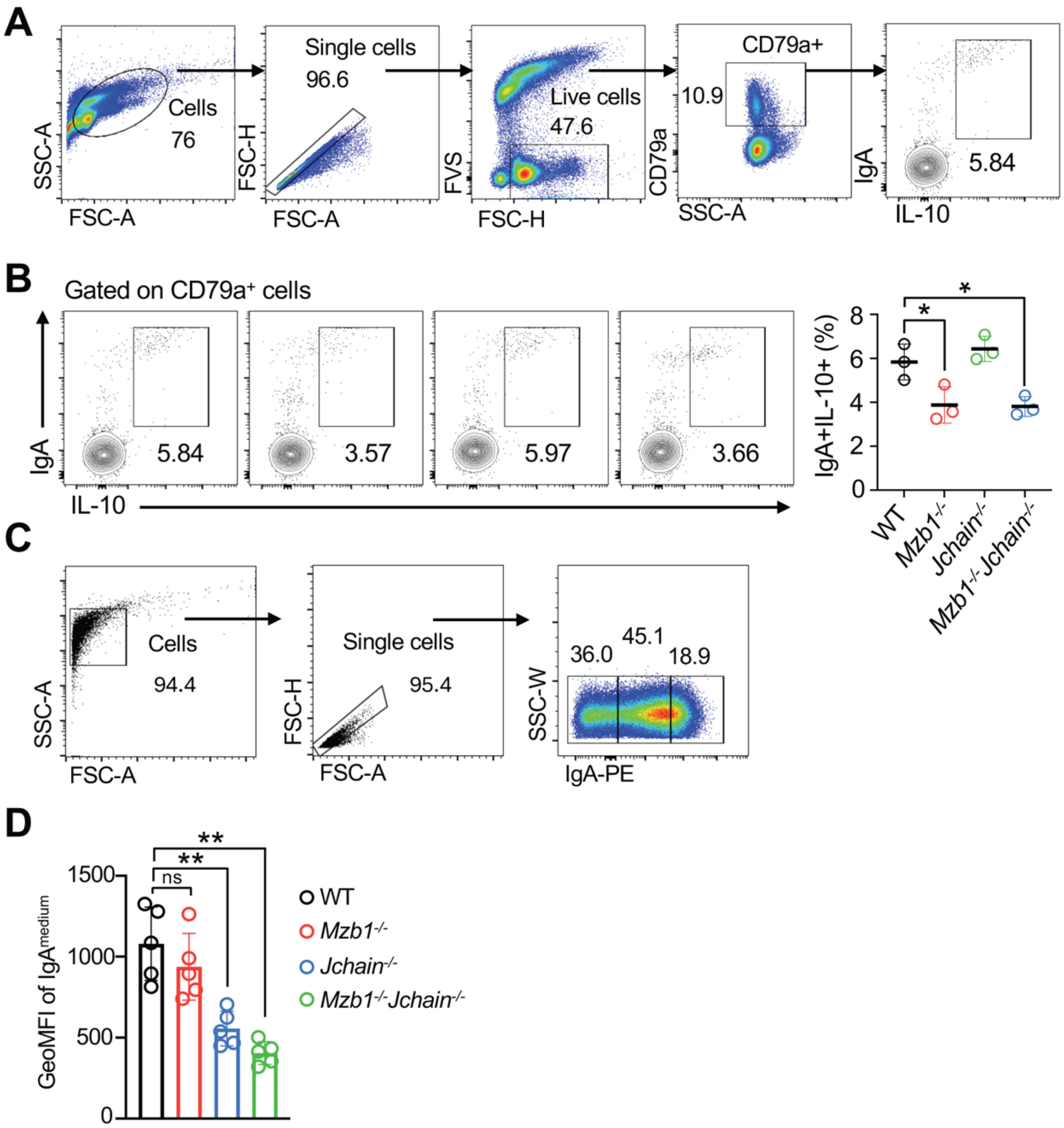

Supplementary Figure 4. Regulatory IgA⁺ PB/PCs and gating strategies.
(**A**) Representative gating strategies for regulatory PB/PCs (CD79a⁺IgA⁺IL-10⁺). (**B**) Frequencies of regulatory PB/PCs in the colonic lamina propria. (**C**) Representative gating strategy for IgA-coated bacteria used in **Figure 5E**.(**D**) Geometric mean fluorescence intensity (GeoMFI) of the IgA^{medium} population shown in **Figure 5E**.
