# Supplementary material for: Cooperative control of IgA synthesis and secretion by MZB1 and the J chain
Source: Front Immunol. 2026 Apr 17;17:1744147. doi: 10.3389/fimmu.2026.1744147 (PMC13132693; doi:10.3389/fimmu.2026.1744147)

Uncropped western blots

Figure 1A

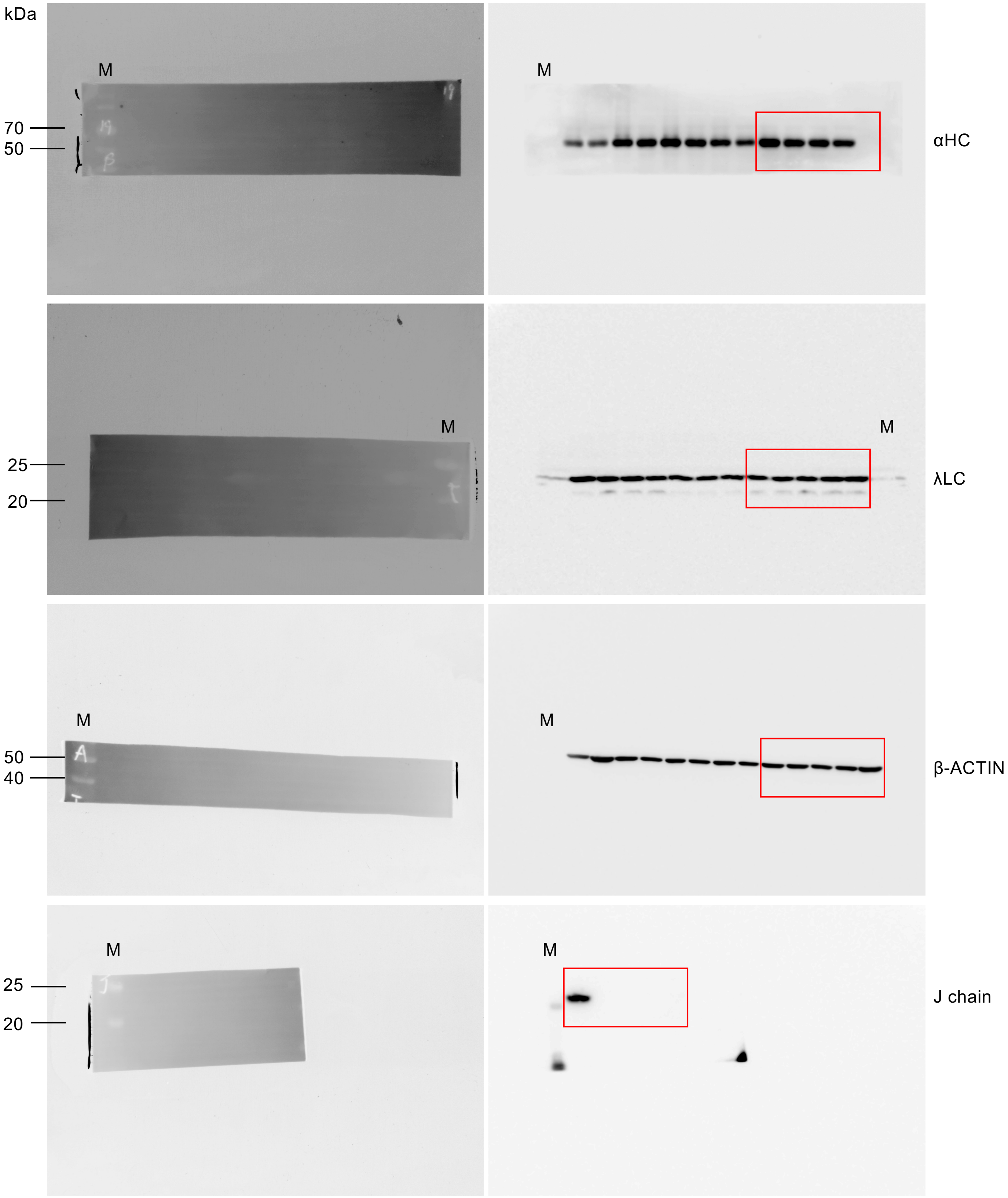

M, molecular weight marker.

Figure 1D, left panel

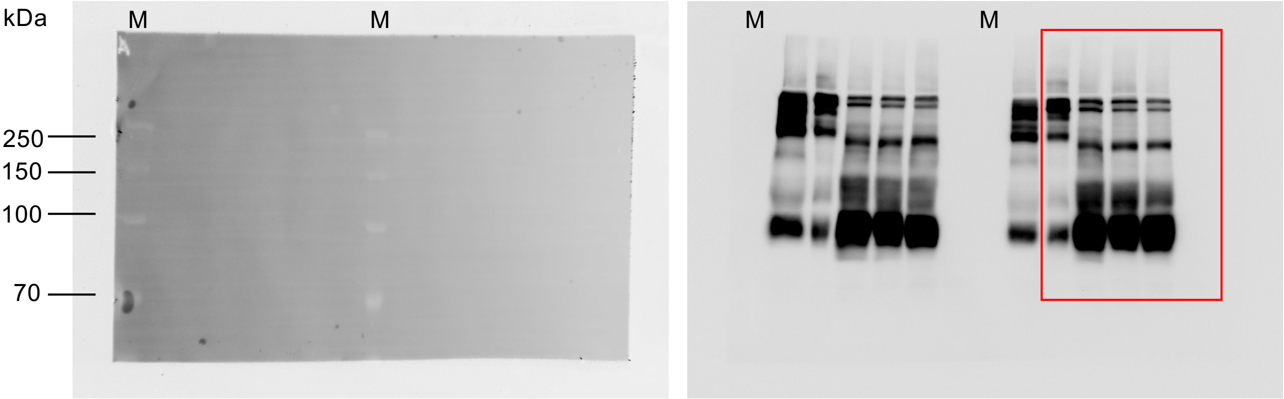

Non-reducing Anti-αHC

Figure 1D, right panel

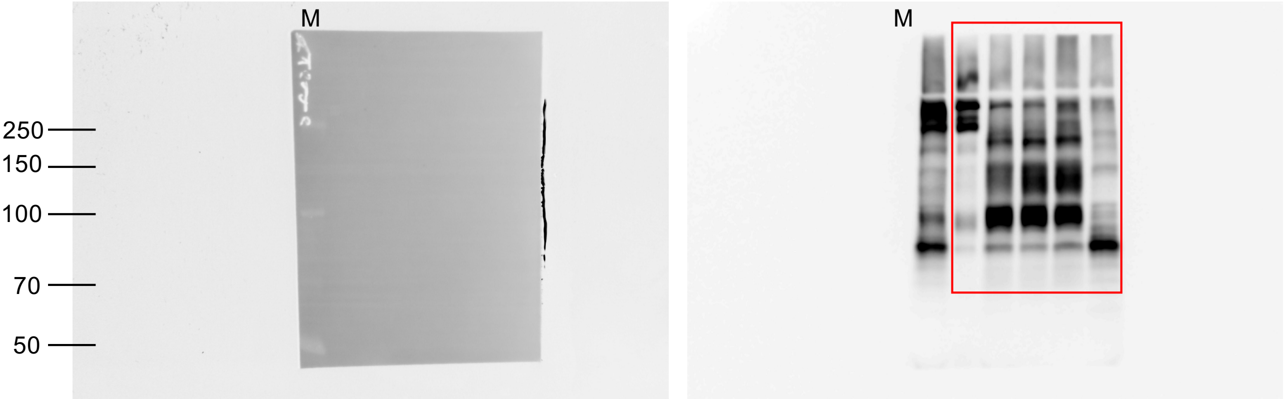

Non-reducing Anti-λLC

Figure 1E, left panel

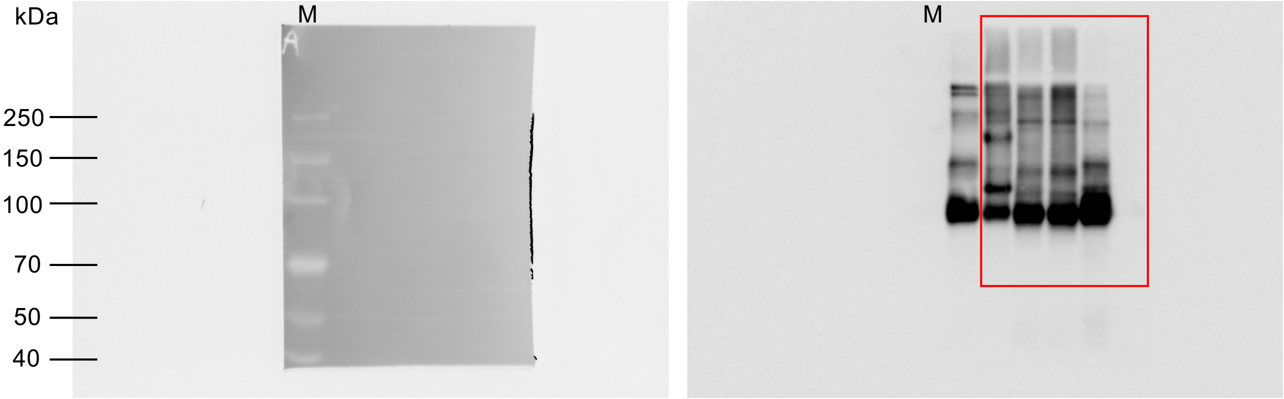

Non-reducing Anti-αHC

Figure 1E, right panel

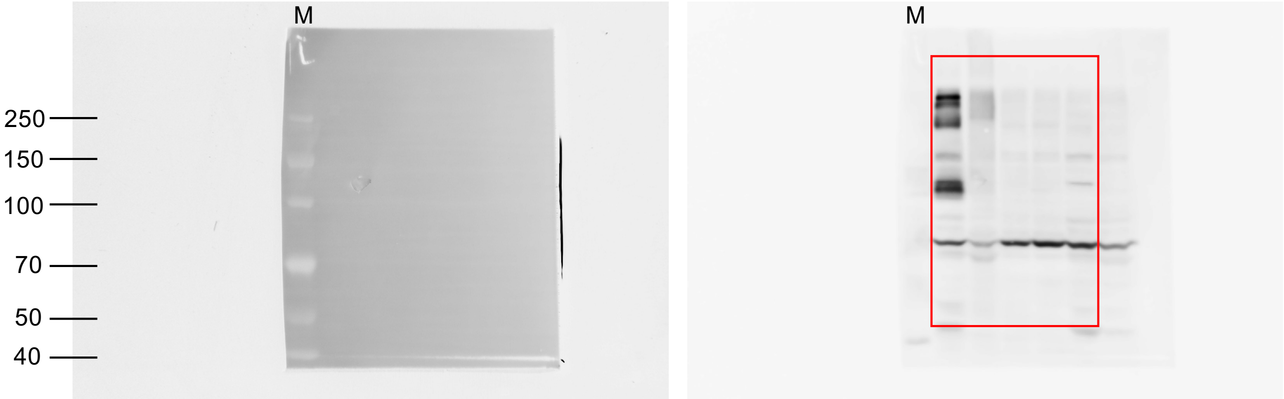

Non-reducing Anti-J chain

Figure 1F

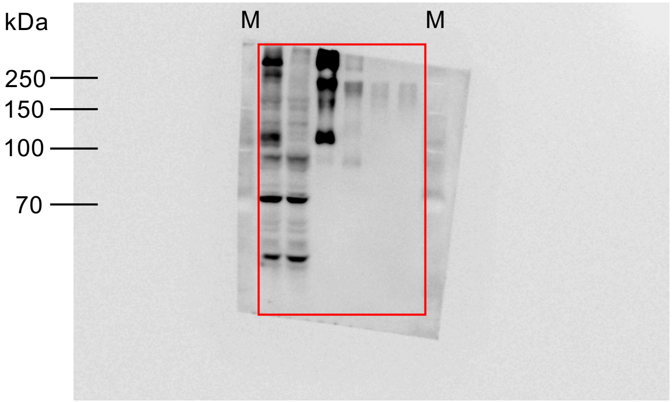

Non-reducing Anti-J chain

Figure 1H

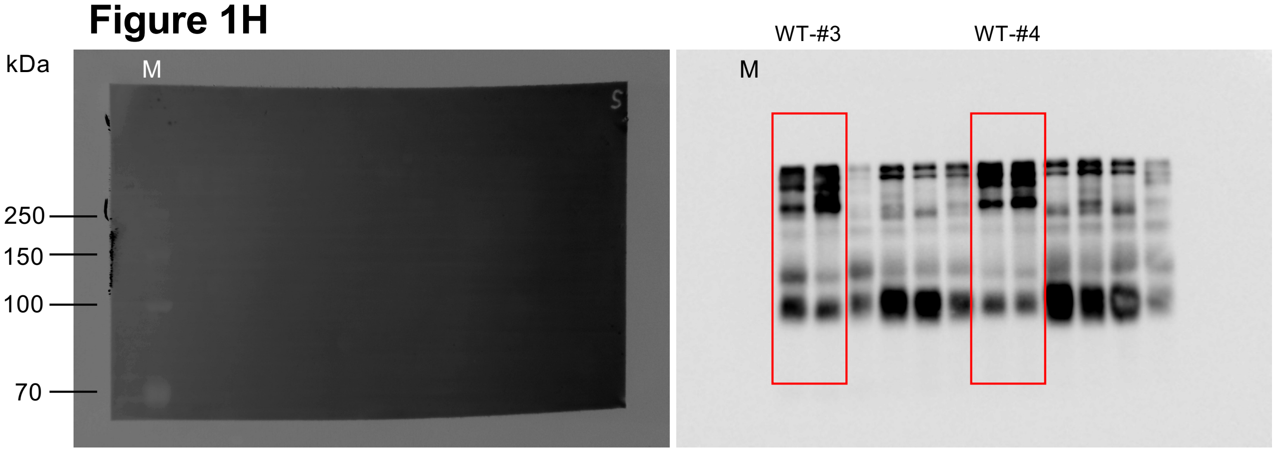

Non-reducing Anti-αHC

Figure 1I

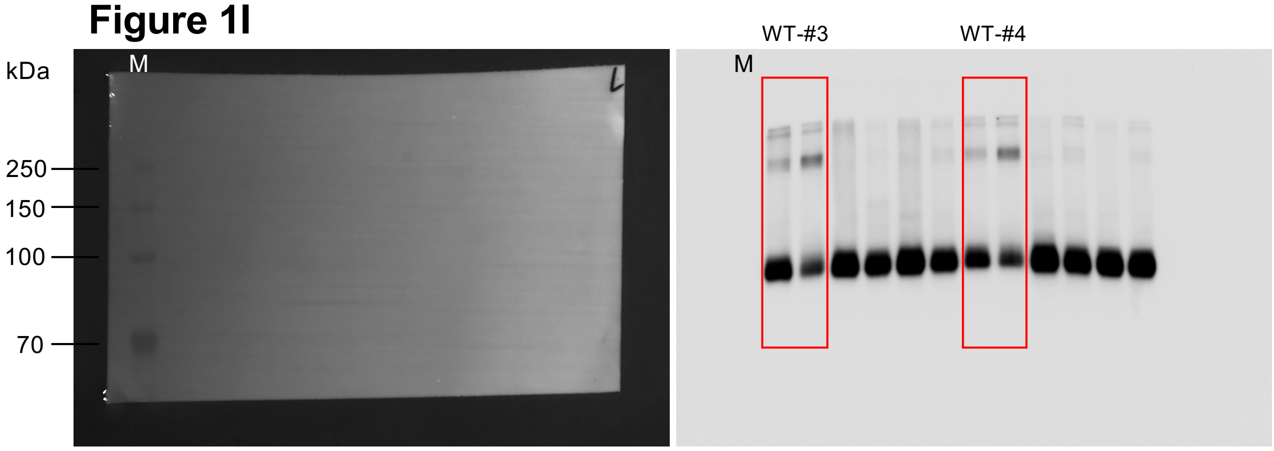

Non-reducing Anti-αHC

Figure 2B

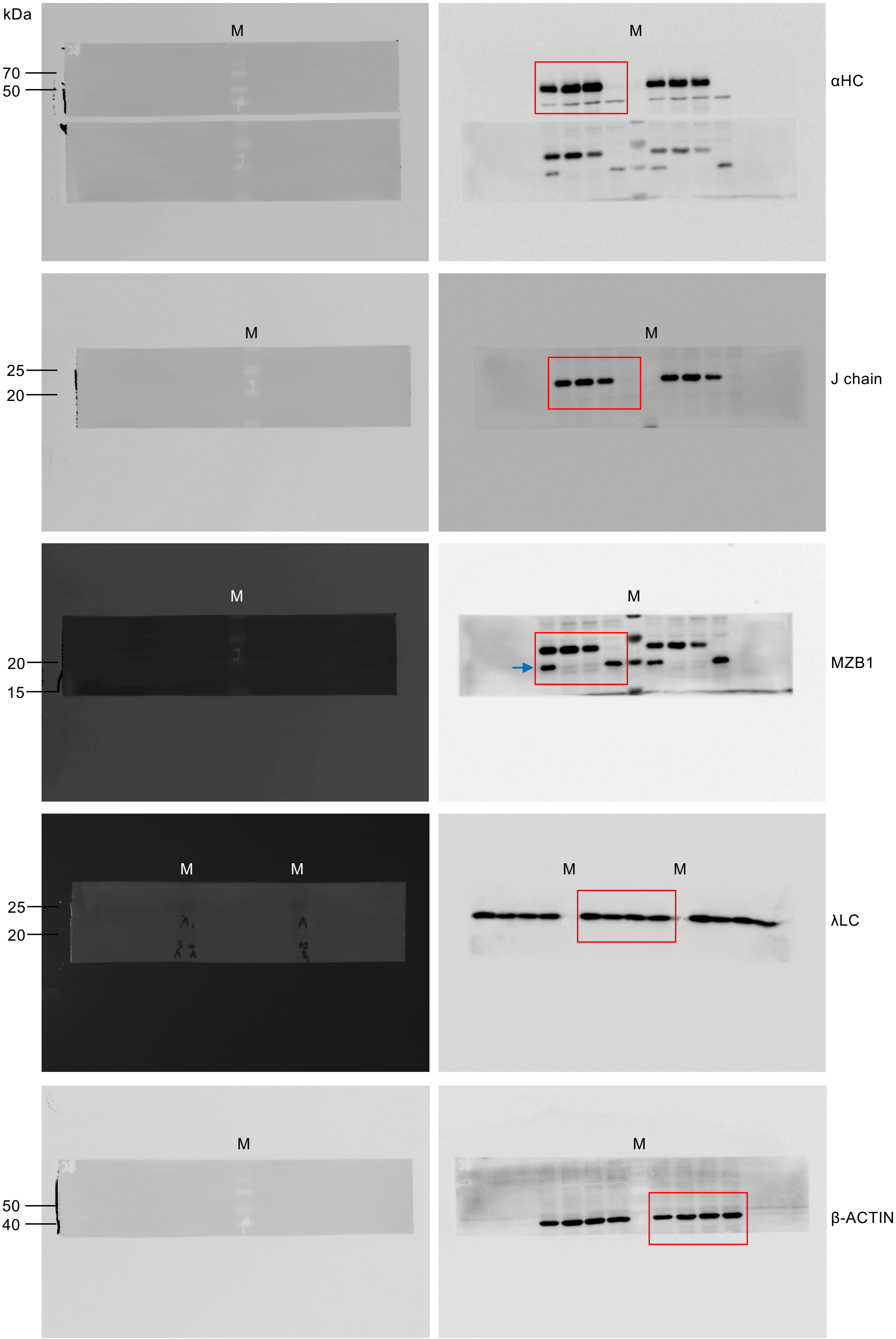

kDa
70
50
M
M
αHC
M
25
20
M
J chain
M
20
15
M
MZB1
M
M
25
20
M
M
λLC
M
50
40
M
β-ACTIN

Figure 2F

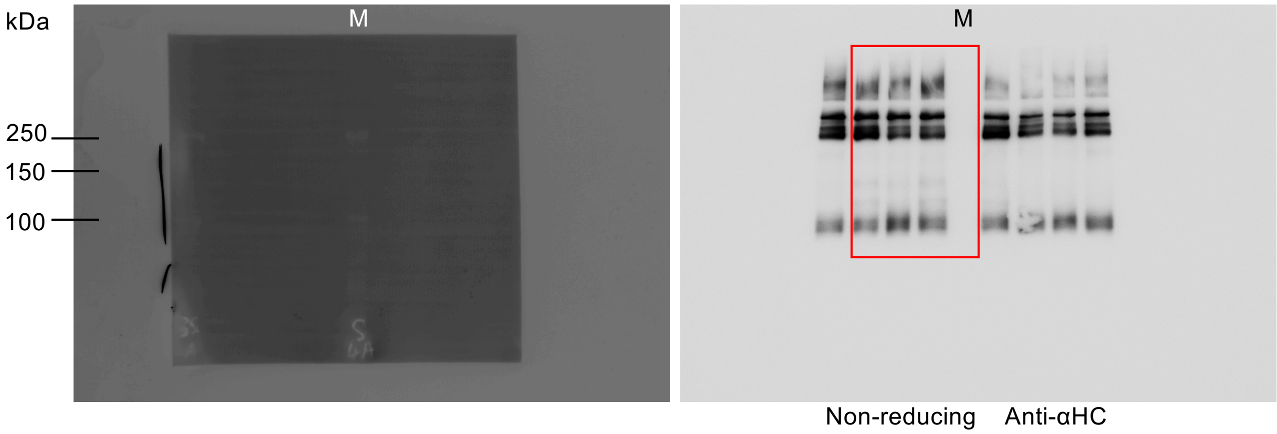

Figure 2G

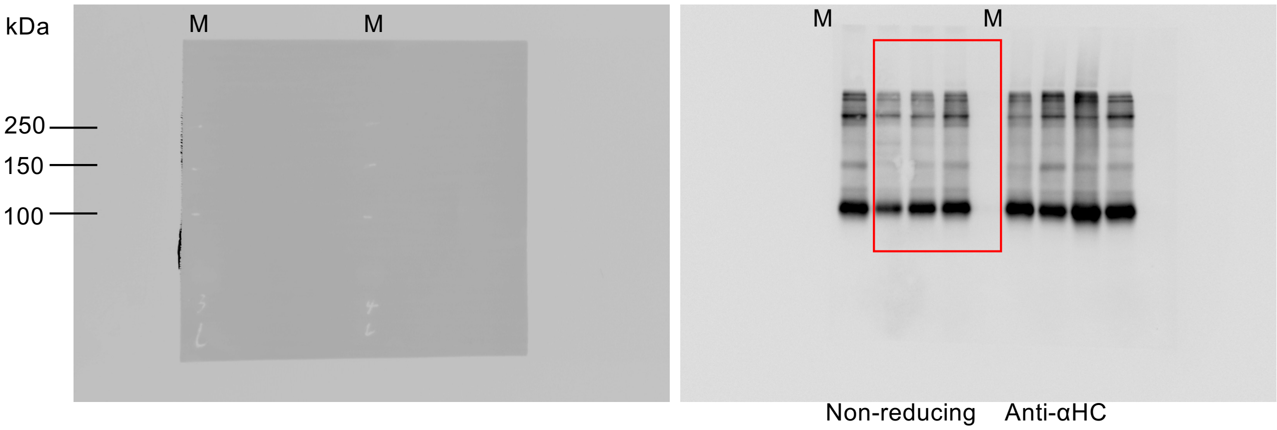

Figure 2I

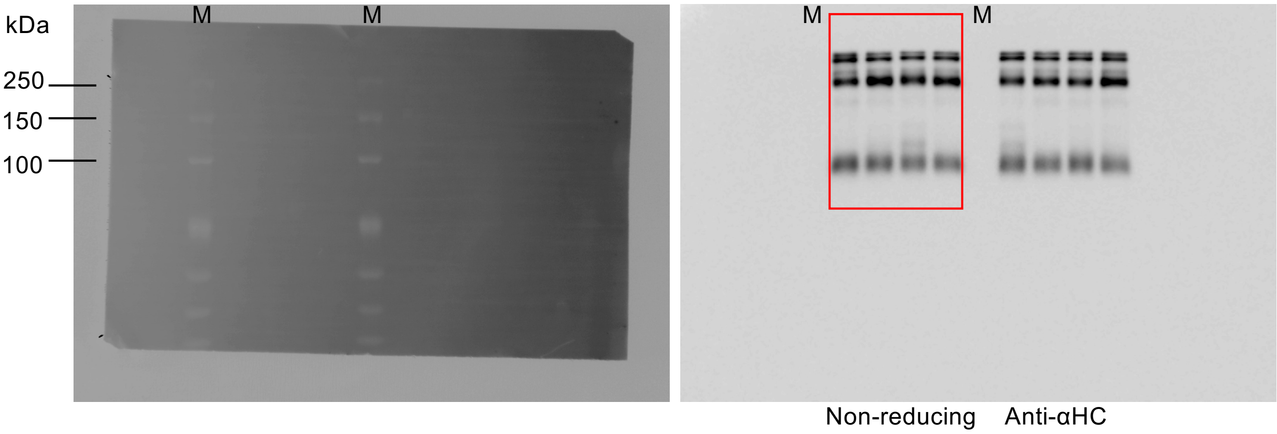

Figure 2J

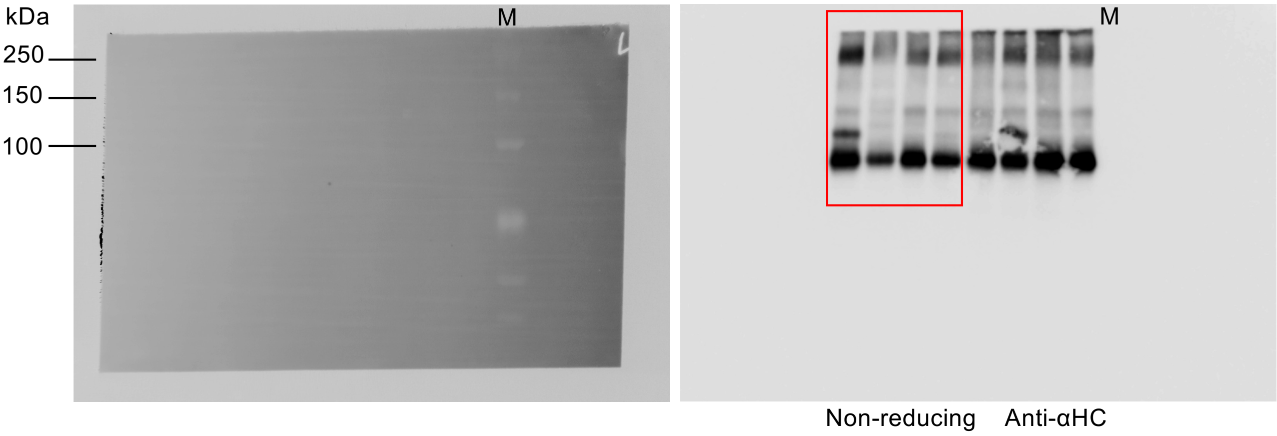

Figure 3A

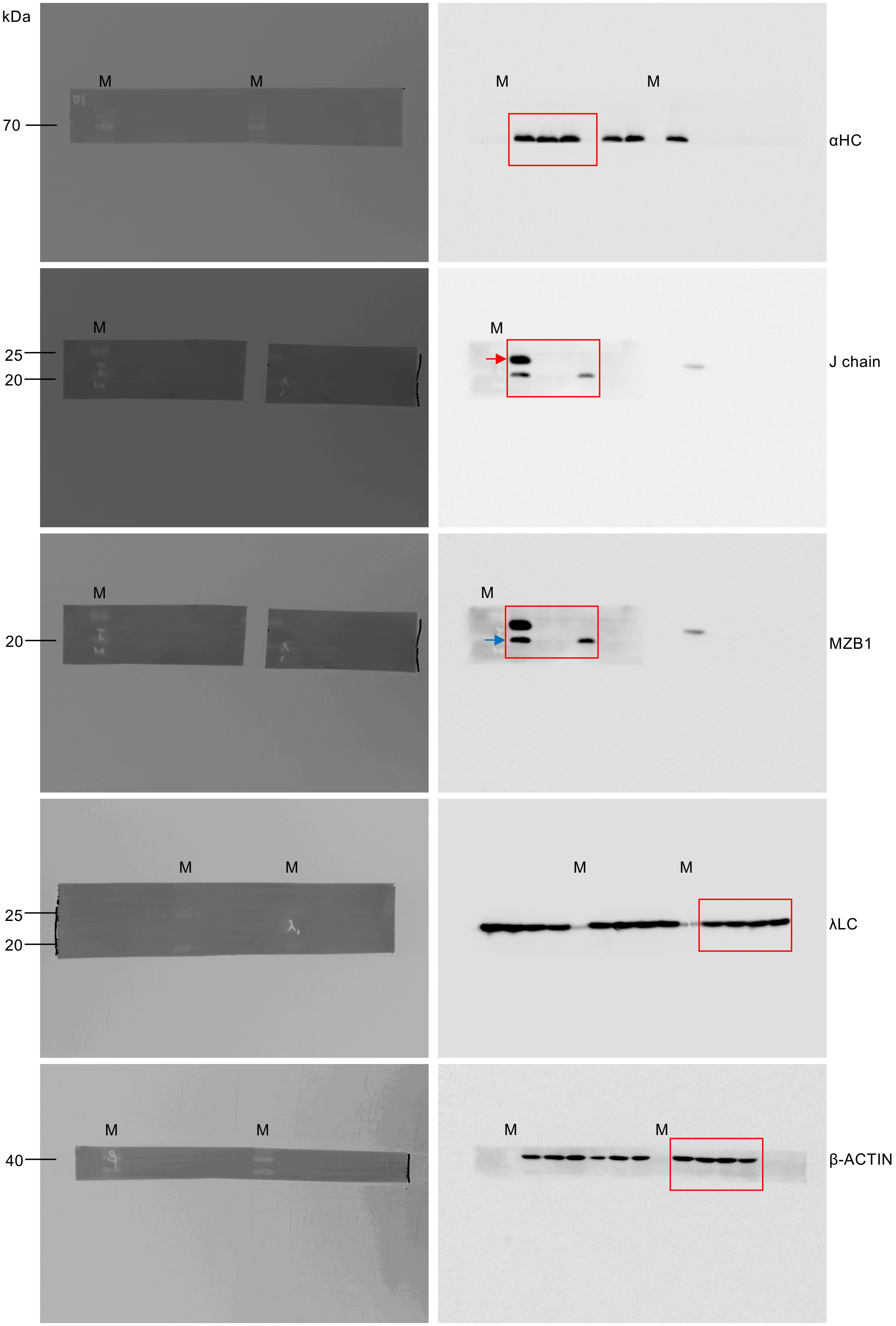

J chain and MZB1 were detected on the same membrane using a mixture of the two primary antibodies and a shared secondary antibody.

Figure 3E

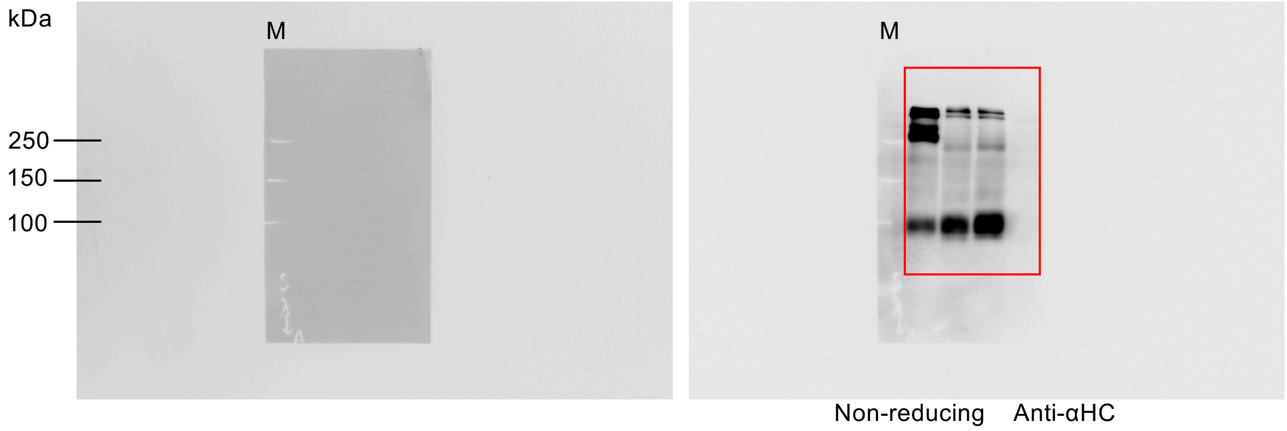

Figure 3F

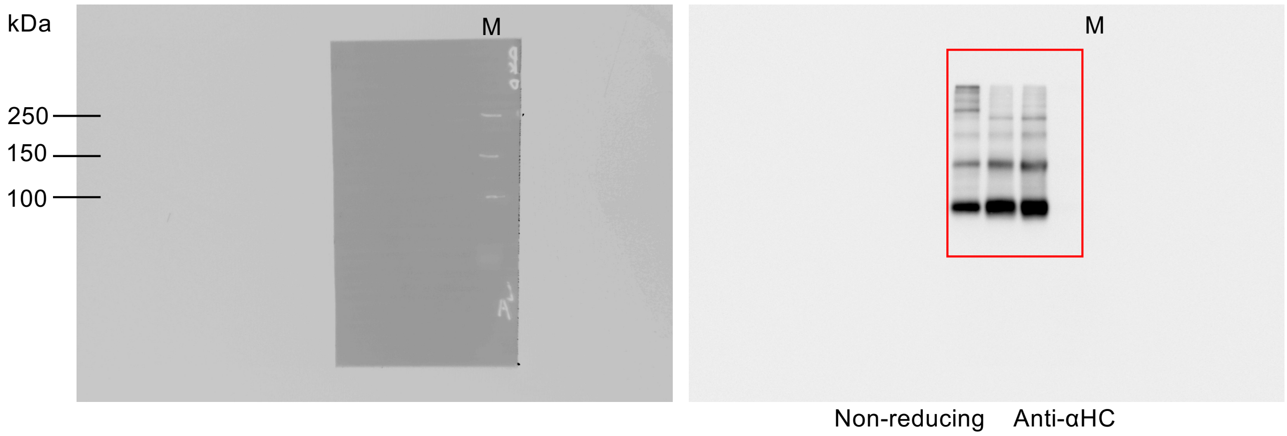

Figure 5B, left panel

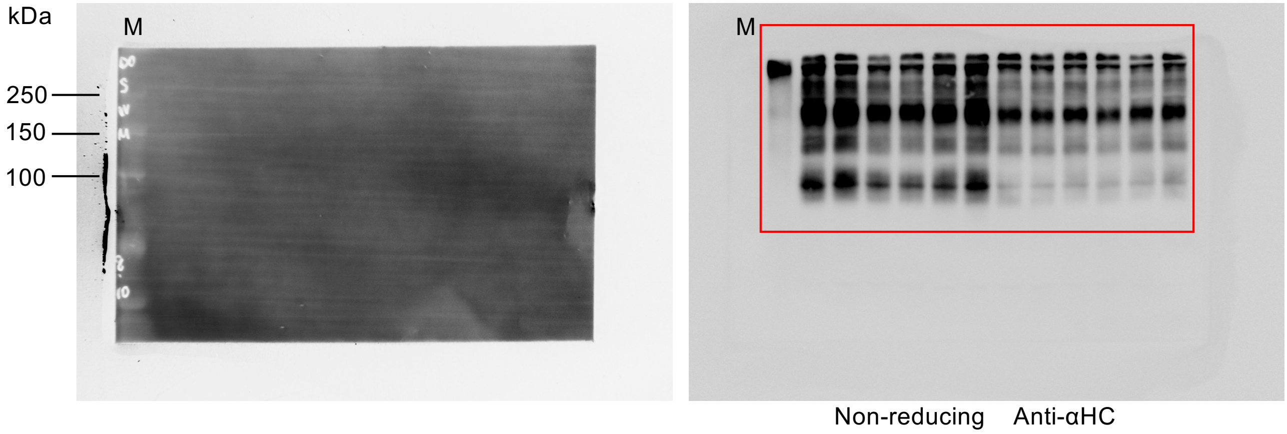

Figure 5B, right panel

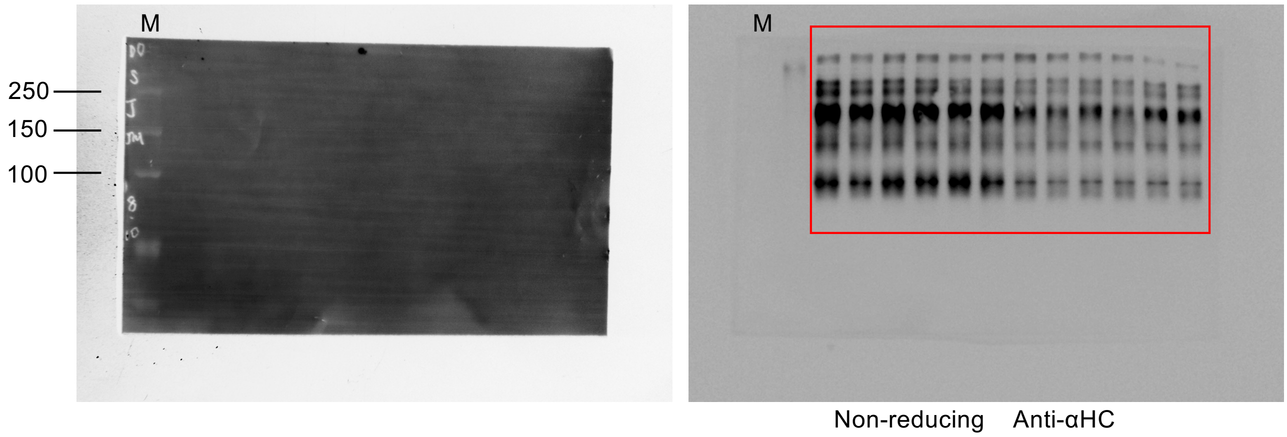

Figure 5D, left panel

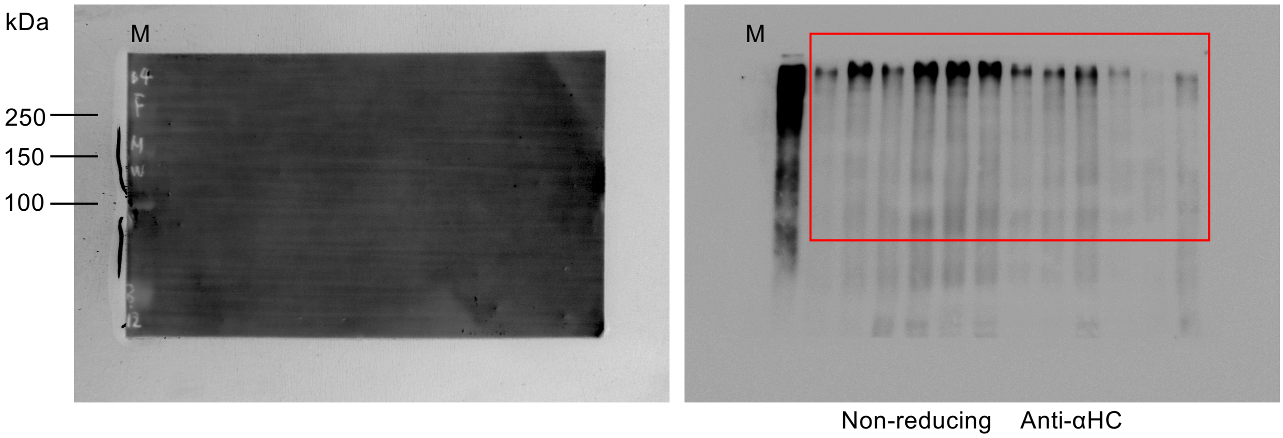

Figure 5D, right panel

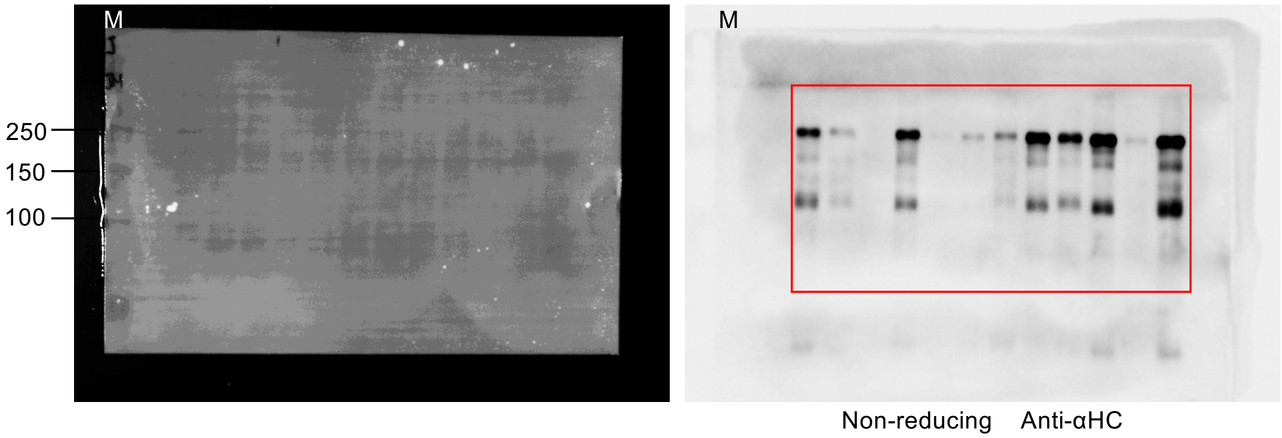

Supplementary Figure 1C

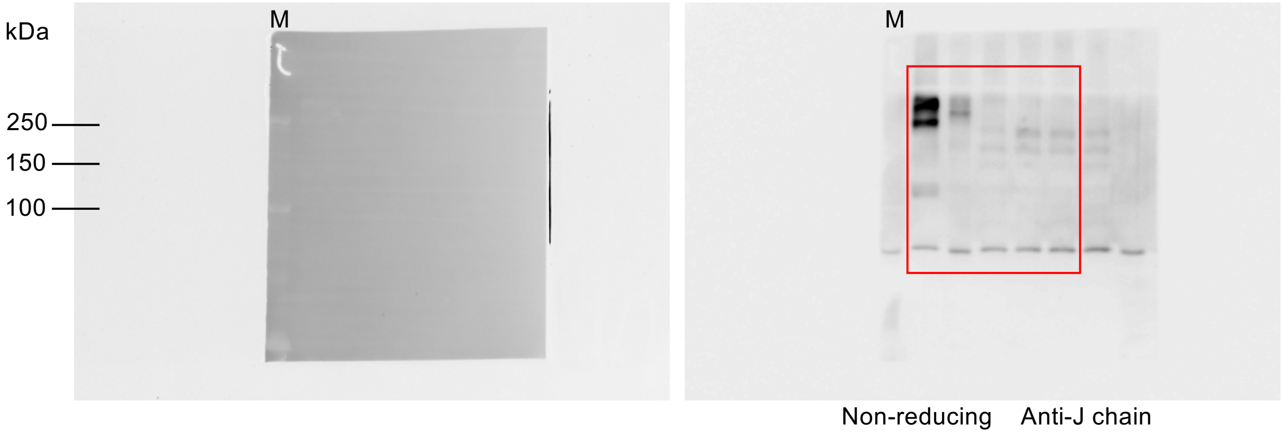

Supplementary Figure 1D

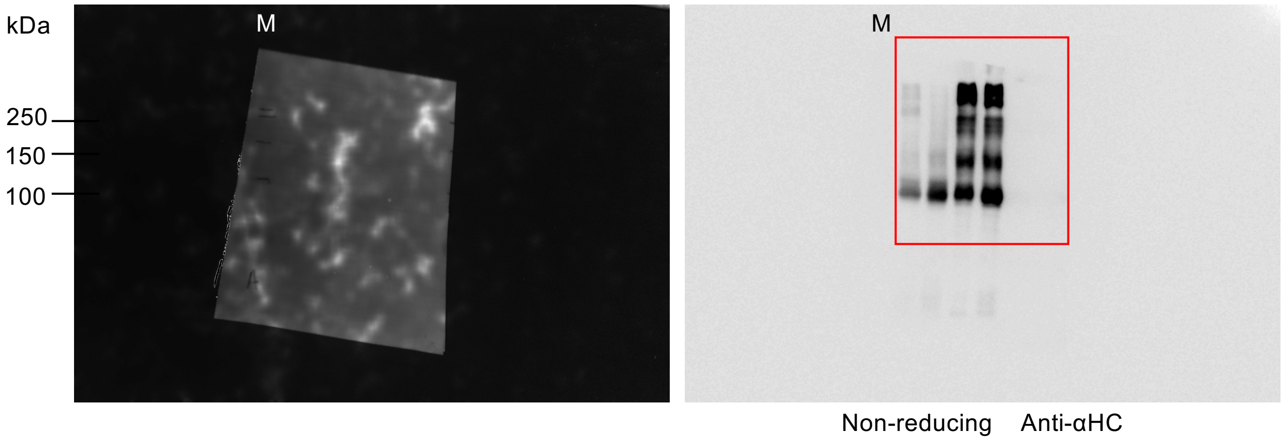

Supplementary Figure 1F

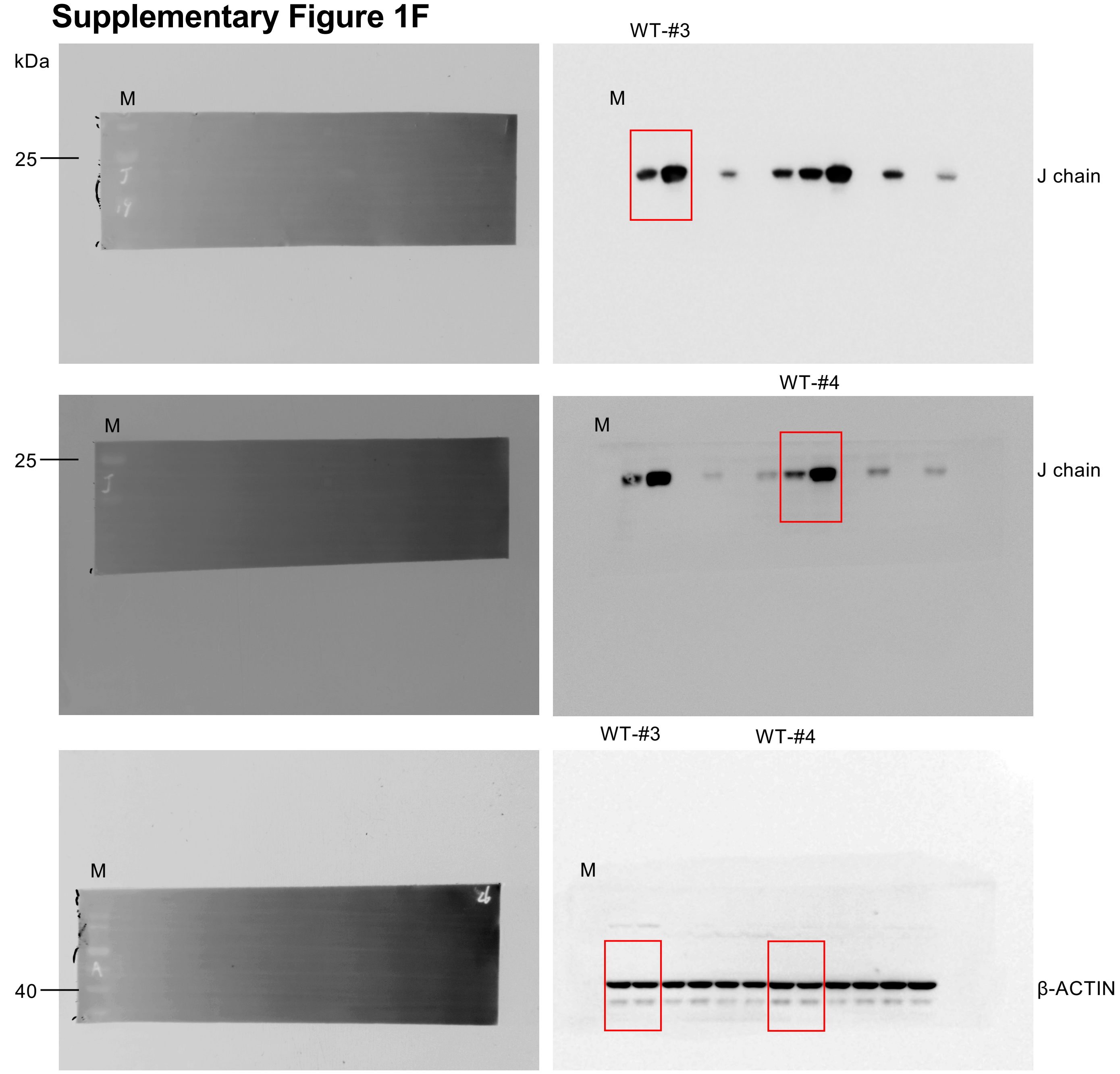

Supplementary Figure 1G

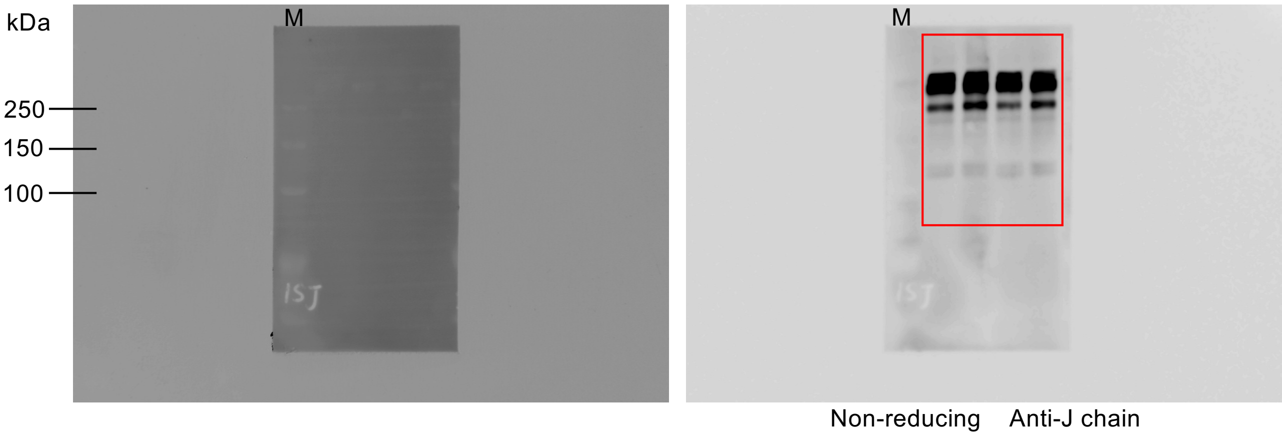

Supplementary Figure 2C

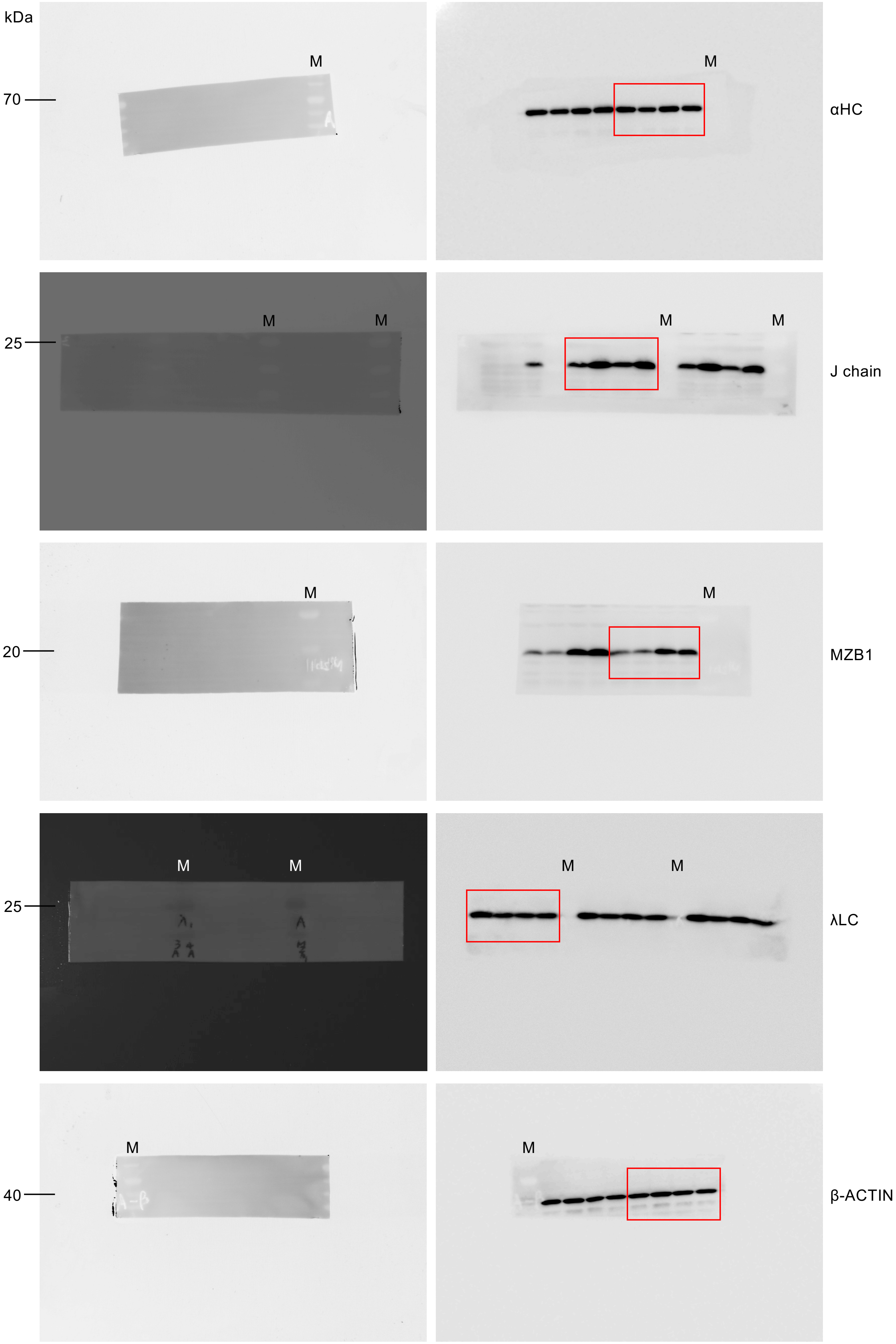

Supplement: Supplementary file 2 [file DataSheet2.pdf]
